# Evaluating the impact of decentralized testing for tuberculosis in Ghana: A simulation model

**DOI:** 10.1371/journal.pgph.0005302

**Published:** 2025-11-17

**Authors:** Parastu Kasaie, Lad Dombrowski, Jeffrey Pennington, Ernest Kenu, Yaw Adusi-Poku, Bernard Wadie, David W. Dowdy, Lee F. Schroeder

**Affiliations:** 1 Department of Epidemiology, Johns Hopkins University, Baltimore, Maryland, United States of America; 2 Department of Pathology, University of Michigan, Ann Arbor, Michigan, United States of America; 3 School of Public Health, University of Ghana, Accra, Ghana; 4 National Tuberculosis Programme, Ghana Health Service, Accra, Ghana; University of California San Francisco, UNITED STATES OF AMERICA

## Abstract

Poor geographical accessibility to tuberculosis (TB) diagnostic services in Ghana is a key barrier to timely diagnosis. Decentralizing molecular testing for TB could improve TB case notification among underserved populations and enhance treatment initiation while avoiding unnecessary treatments. This study aims to project the epidemiological impact of decentralized TB testing at the country level. We developed an individual-based simulation of the TB diagnostic system and treatment cascade in Ghana, calibrated to key TB care targets using data from the District Health Information Management System (DHIMSII) from 2019-2022. We assessed the effects of expanding molecular (Xpert MTB/RIF) testing to (1) all 218 district hospitals and (2) all 703 modeled facilities reporting TB notifications, comparing these scenarios to the current diagnostic network. Under the baseline scenario, 26% of samples remained untested, with over 30% of diagnoses made through clinical judgment alone, and 35% with false positive diagnoses. Decentralizing Xpert to district-level facilities could reduce the proportion of untested samples to 20%, lowering false positive diagnosis to 32%. While projected TB notifications and treatment rates in 2030 remained similar, this shift could result in 51 additional treatment initiations for individuals with undiagnosed TB, prevent 36 unnecessary treatments, and save 43 lives per million people over an eight-year period by 2030. Full Xpert decentralization could eliminate untested samples by enabling on site testing at all facilities, reduce false positive diagnosis to 25%, and lead to 220 additional treatment initiations, 140 fewer unnecessary treatments, and 180 lives saved per million relative to baseline from 2023-2030. Decentralizing Xpert testing offers significant benefits, improving diagnostic accuracy, reducing untested samples, and enhancing TB treatment outcomes – but wide-scale implementation is required to realize full benefits at a country level.

## Introduction

Tuberculosis (TB) remains a significant public health challenge in Ghana, posing a substantial economic and health burden. A major contributor to this burden is underdiagnosis; for example, of the 44,000 people in Ghana estimated to develop incident TB in 2020, only 13,000 (37%) were notified to public health authorities [1]. Improving TB diagnosis in Ghana is therefore a key health priority.

One of the main barriers to timely TB diagnosis in Ghana is poor geographical accessibility to diagnostic services [2,3]. TB diagnostic services primarily occur at government health facilities in the districts or at regional hospitals. This poses an obstacle for rural residents and some private health facility attendants who must travel to, or have specimens sent to, these central facilities for confirmatory tests, rather than local health centers and some private health facilities which would be more accessible geographically [4,5]. As of 2021, less than 6% of health facilities in six Ghanaian regions – Upper West, Upper East, Northern, North-East, Ahafo, and Savannah – were providing TB diagnosis services [5]. The estimated mean distance to nearest TB diagnosis site was 23 km, corresponding to a traveling time of over 60 minutes, but estimates vary significantly across the regions. Rural populations in particular face additional travel barriers, including limited transportation options and poor road conditions. As in many other low-resource settings, limited access to diagnostic facilities and long travel distances remain key health system-related barriers to timely TB case detection in Ghana [4,6,7].

Diagnostic accuracy also plays a crucial role in improving TB notification. Sputum smear microscopy, historically the primary diagnostic method, has imperfect sensitivity, resulting in missed diagnoses [2,3]. Although molecular diagnosis with Xpert Ultra (“Xpert”) has replaced smear microscopy in recent years, its availability remains limited and uneven across regions, with over 30% of people with TB in Ghana still being diagnosed clinically without bacteriological confirmation [5,8]. As of 2023, GeneXpert instruments had been distributed to over 170 health facilities across the country, accounting for 24% of all facilities reporting any TB diagnosis in the preceding three years. Furthermore, over 90% of Xpert-equipped laboratories are concentrated at secondary and tertiary care levels, limiting the availability of TB diagnostic services in sub-district health centers and local clinics [5,9]. This requires patients presenting with TB symptoms to travel to the higher-tier health facilities to confirm their diagnosis, or to have sputum samples sent through specimen transport networks that face logistical challenges such as specimen degradation and/or loss [10,11]. While there has been efforts to establish a centralized transportation system to support sample transfer from hard-to-reach areas without a testing site to designated facilities equipped with molecular testing [5,12], this strategy is not currently implemented uniformly across regions.

These geographical accessibility issues for TB diagnosis in Ghana reflect broader systematic challenges in delivery of primary health care in many low- and middle-income countries. In response to existing gaps, the Ghana Health Services and the Ministry of Health have developed the “Network of Practice” (NoP) strategy, aiming to increase access to quality health services for all Ghanaians by 2030 [13]. The NoP model follows a hub-and-spoke structure aimed at optimizing resource distribution and service delivery at a local level. In this model, health centers serve as “hubs”, providing technical and operations supports to nearby “spokes”, which include community-based health planning and services (CHPS) facilities and in some cases other health centers. This network structure has shown to enhance mutual technical and operational support among connected facilities, thereby expanding the scope of service delivery within the network [14]. This strategy also includes upgrading selected hubs into “Model Health Centers”, delivering higher standards of care with improved efficiency. These centers are adequately equipped to prevent, diagnose and manage communicable diseases such as malaria, HIV and TB.

Here, we focus specifically on the laboratory-based diagnosis of TB in Ghana, with an emphasis on the question of how best to offer decentralized molecular diagnostic testing with Xpert within the NoP model. Decentralizing Xpert testing to all district hospitals has the potential to streamline the diagnostic process, reduce transportation-related delays, and improve the overall efficiency of TB diagnosis and treatment initiation [5]. Further extending Xpert availability at the sub-district level – to health centers and selected CHPS facilities – could bring diagnostic services even closer to where people work and live. This strategy would be especially impactful for remote and underserved communities. However, while these scenarios offer clear benefits for expanding access and improving equity in TB diagnosis, their feasibility remains uncertain. Beside the cost of GeneXpert instruments, successful implementation would require substantial investments in infrastructure, skilled workers, equipment maintenance and reliable supply chain for the test components.

The NoP strategy offers a practical and scalable framework for addressing these challenges in the coming years. Through its hub-and-spoke structure, NoP enables strategic placement of Xpert machines at designated hubs – particularly Model Health Centers – equipped with necessary infrastructure and laboratory capacity to operate and maintain the equipment. These hubs not only serve as testing sites, but also provide technical support to connected spoke facilities, helping to ensure proper sample collection and referral procedures from peripheral sites. Moreover, the NoP supports logistical coordination within the networks, facilitating the sputum sample transportation from spoke sites network of Xpert-equipped hubs. This integrated approach can increase access to timely and accurate TB diagnosis, thereby improving TB notification and treatment initiation, and ultimately reducing ongoing transmission, morbidity, and mortality [5,9,15,16].

As Ghana moves toward Universal Health Coverage, improving geographical accessibility to diagnostic services is a timely and essential topic to explore – one that will shape the future of TB control and other communicable diseases in Ghana. To guide future decision making, it is important to quantify the potential impact of expanding Xpert coverage – in terms of additional TB notifications, treatment initiations, and reductions in TB-related deaths. To this end, we utilized an individual-based model of the TB diagnostic system and treatment cascade in Ghana to evaluate the effects of expanding Xpert testing to 1) all district hospitals or 2) all facilities reporting TB cases, compared to the current centralized system.

## Materials and methods

We developed an individual-based simulation model to assess the TB diagnostic system and treatment cascade in Ghana, using data from the District Health Information Management System (DHIMSII), an integrated electronic database capturing health data across Ghana’s healthcare system [8]. The model incorporates TB screening and case registration results from DHIMSII from 2019-2022 and focuses on evaluating the impact of alternative TB diagnostic placements.

### TB diagnostic network structure

The TB diagnostic network in Ghana is organized into seven tiers, representing the roles of different facilities during the calibration period from 2019 to 2022 (**Fig A in S1 File**). Tiers 1 and 2 include the National and Zonal Public Health Reference Laboratories offering only testing services (without clinical services for patient notification and treatment). Tier 3 encompasses Regional Hospitals, offering both testing and clinical services. District Hospitals (Tier 4); Hospitals (Tier 5); Health Centers (Tier 6); and Community Health and Planning Stations (CHPS) and Clinics (Tier 7) have varying testing capabilities, dependent on available resources. In facilities without in-house Xpert testing (including the majority of those in Tier 5–7 and some in Tier 4), samples are transported to closest facilities equipped with Xpert, or patients may choose to self-refer.

Our model includes all facilities that notified at least one person with TB between 2019 and 2022. We also include all district hospitals, existing Xpert sites, and facilities designated for a future Xpert expansion according to government plans, even if they did not notify any cases of TB during that four-year period. In total, our model includes 703 health facilities, of which 170 were designated as Xpert sites at baseline (**Table A in** **S1 File**).

### Characterizing population catchment areas for facilities

We assumed that patients present to the nearest facility based on the shortest travel time from their residence for diagnostic and treatment services. Each facility in the model is linked to a catchment area representing the population closest to it. Using AccessMod (5.6.0) and ArcGIS Pro (3.2.0), we estimated these catchment areas by calculating the shortest travel times to each facility, incorporating terrain, population, and infrastructure data tailored to Ghana’s geography [17,18]. Each catchment area was further divided into ten concentric bands based on travel times to represent varying patient travel distances (**Section A.3 in** **S1 File**).

### Estimating the population size of individuals with presumed TB infection

The model simulated populations of individuals with TB and those without TB but experiencing TB-like symptoms. The number of individuals with TB presenting at each facility was proportional to the estimated number of incident TB cases in each geographic area, accounting for population size and the TB case notification ratio [1,19]. Spatial modeling was carried via AccessMod, which relies on population distribution data from WorldPop [20]. However, because WorldPop data is only available through 2020, we used the population trend from Macrotrends to extend estimates to 2030 [19] (**Table B in** **S1 File**). Finally, we estimated the number of persons without TB but presenting with TB-like symptoms to be proportional to the number of TB cases presenting at each facility.

### Simulated patient pathway

**Fig 1:**
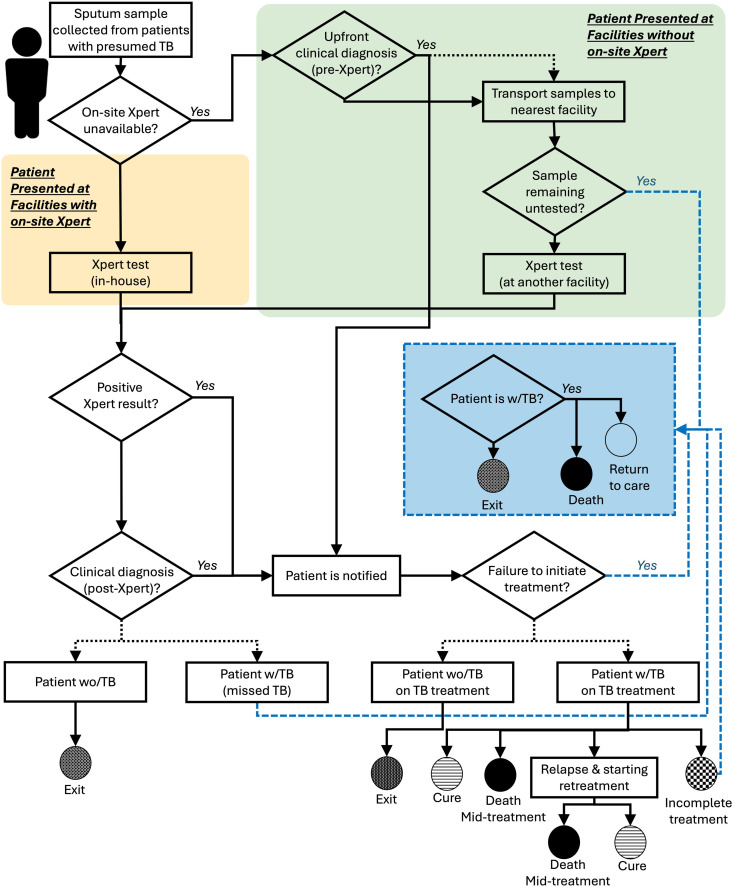
Tuberculosis (TB) diagnosis and treatment pathway for simulated patients. The model simulates patients presenting with TB-like symptoms who are presumed to have TB and undergo sputum sample collection. This includes patients with (w/) and without (wo/) TB infection. If on-site Xpert is available at the facility that the patient present to (yellow box), testing is carried out in-house and same-day diagnosis is provided. Alternatively, if Xpert testing is unavailable at the facility (green box), patients experience a probability of receiving an “up-front clinical” diagnosis prior to Xpert results being received, allowing for immediate notification and treatment initiation. All sputum samples are then transported to the nearest facility with testing capacity, however, a certain proportion of samples may remain untested. A positive Xpert result confirms a TB diagnosis. Negative Xpert results are further reviewed by clinicians who can still make a clinical diagnosis despite negative Xpert results. Patients diagnosed with TB (based on a positive Xpert test or a clinical diagnosis) are notified and typically initiate treatment. However, a certain proportion of diagnosed patients may fail to initiate treatment. Patients receiving treatment for TB may experience one of several outcomes: recover, fail to complete treatment, die during the course of therapy or experience relapse and start retreatment for TB. Diamonds represent decision points in the system. The model includes several pathways through which patients with TB may remain untreated (dashed blue lines), potentially resulting in death or future re-engagement with the care system.

Our model tracks patients who arrive at a health facility with TB-like symptoms, who are presumed to have TB, and undergo sputum sample collection (**Fig 1**). If TB testing is available in-house, the sample is tested in-house, and the patient receives a same-day diagnosis. Alternatively, if Xpert testing is unavailable at the facility, patients experience a probability of receiving an “up-front clinical” diagnosis, allowing for immediate notification and treatment initiation. Following this, all collected samples are transported to the nearest facility with testing capacity. However, a certain proportion of samples may remain untested due to various factors, including issues with sputum collection, sample transport, lack of reagent, instrument out-of-service, error flags, or workforce issues. Of note, although Xpert testing is ordered for patients receiving up-front clinical diagnosis, Xpert results do not impact treatment decisions in this case.

Following potential testing delays and after any failures in testing during the sent-out process, Xpert results are relayed to clinicians. We assume that all positive Xpert results lead to a TB diagnosis, while patients with negative Xpert results may still be clinically diagnosed with TB based on symptoms.

Patients diagnosed with TB initiate treatment at the nearest facility, however, a certain proportion may remain diagnosed but do not receive treatment. While most patients recover, some may not complete treatment, die during treatment, or fail treatment, at which point they reinitiate therapy. Treatment outcomes among people without TB are not tracked in the model.

### Simulated diagnostic strategies

We considered three diagnostic strategies, each reflecting different approaches to Xpert testing placement in Ghana’s healthcare system:

- **Current Xpert Placement (Baseline as of 2023)**: Xpert testing is available at 170 selected facilities, including national and zonal public health reference laboratories, regional hospitals, 122 district hospitals, 24 non-district hospitals, and 4 health centers and 2 CHPS/clinics (**Table A in** **S1 File**). Facilities without Xpert testing rely on sample transport to closes facilities equipped with Xpert.
- **District-level Decentralization:** Additional GeneXpert instruments are distributed to all 218 district hospitals, bringing the total number of Xpert-equipped facilities to 266. This reduces the need for sample transport for patients accessing care at these larger, district-level facilities.
- **Full Decentralization:** GeneXpert instruments are distributed to all 703 modelled facilities, including hospitals, health centers, and CHPS/clinics report TB notifications. This scenario eliminates the need for sample transport and minimizing the risk of samples remaining untested. This scenario represents an idealized configuration to establish a ceiling on diagnostic gains that could be realized with increasing geographical access to TB testing across healthcare system.

### Calibration

The calibration methodology involved iteratively adjusting the model’s input parameters to minimize discrepancies between simulated results and observed TB outcomes in Ghana. To achieve this, we first estimated a set of prior distributions for all simulation parameters based on existing data (**Table 1 and Table C in** **S1 File**) [8,21–25]. Next, we identified four calibration targets, representing key aspects of TB care in Ghana: 1) the number of people presumed to have TB upon presentation for health care (those who meet clinical criteria), 2) the number of TB tests performed, 3) the number of TB diagnoses, and 4) the proportion of clinically diagnosed pulmonary TB notifications (those that do not have laboratory confirmation of disease) (**Table D in** **S1 File**).

**Table 1:** Model parameters. Prior distributions are represented as the median (2·5-97·5 percentile) for beta distributions, and as [minimum – maximum] for uniformly distributed priors. Uniform priors are chosen to be minimally informative, and their ranges were adjusted empirically such that well-fitting models had values far from the endpoints.

Parameter	Prior distribution (mean) [Range]	Reference
Proportion of people with incident TB presenting to care	Uniform [0.15 – 0.35]	[25]
Number of persons without TB presenting to care, per person with TB	Uniform [14 – 30]	[8]
Proportion of patients with TB clinically diagnosed up-front prior to Xpert	Uniform [0 – 0.3]	[24]
Proportion of patients without TB clinically diagnosed with TB up-front in the absence of Xpert	Uniform [0 – 0.05]	Assumption
Probability of sample remaining untested	Uniform [0 – 1]	[8]
Probability of patient failing to initiate treatment	Beta (0.14) [0.1 – 0.4]	[8,21]
Xpert test sensitivity	Beta (0.85) [0.78 – 0.92]	[22]
Xpert test specificity	Beta (0.99) [0.98 – 1]	[22]
Sensitivity of clinical diagnosis among patients receiving a negative Xpert result for TB	Uniform [0 – 0.3]	[24]
Specificity of clinical diagnosis among patients receiving a negative Xpert result for TB	Uniform [0.99 – 0.999]	Assumption
Probability of recovery following TB treatment	Beta 0.85 [0.63 – 1]	[8]
Probability of incomplete treatment among patients who do not recover after receiving TB treatment	Beta 0.33 [0.2 – 0.46]	[8]
Probability of death during initial TB treatment among patients who do not recover after receiving TB treatment and do not complete treatment	Beta 0.9 [0.5 – 1.0]	[8]
Probability of recovery following TB retreatment	Beta 0.85 [0.63 – 1]	[8]
Time to death during TB retreatment among patients who did not recover	Uniform [0 – 180] days	[40]
Probability of representation to care (per month)	Beta 0.1 [0.05 – 0.15]	Assumption
Probability of death before representation to care	Uniform [0.3 – 0.8]	[8,25]
Time (days) to spontaneous recovery	Uniform [1 – 3600]	Assumption

TB: Tuberculosis.

We used a Bayesian Sampling-Importance-Resampling (SIR) framework to update parameter estimates by sampling from prior distributions and resampling based on how well simulated outcomes matched the calibration targets.[26] To do this, we ran 50,000 simulations for 2021 (the calibration year), using an initial set of prior distributions. Goodness of fit to our calibration targets was assessed using a log-likelihood function. Finally, we resampled 1,000 best-fitting parameter sets to project outcomes to 2030 (**Section B in** **S1 File**).

### Statistical and sensitivity analysis

We conducted a sensitivity analysis on five key outcomes: Xpert tests performed, new TB diagnoses, incorrect diagnoses, TB cures, and TB-related deaths. First, we examined how changes in model parameters affected projected outcomes under the baseline scenario by year 2030 (end of projections). Next, we assessed the sensitivity to model parameters of projected changes in these outcomes when comparing the district-level decentralized scenario to the baseline scenario through 2030. We calculated partial rank correlation coefficients (PRCCs) for each parameter to estimate association with each primary outcome. Parameters with a strong correlation (|PRCC| > 0.1) were selected for a one-way sensitivity analysis in which we compared simulations with the parameter in its highest quartile to those with the parameter in its lowest quartile (using a threshold of +/-25% in the outcome value to identify differences of potential epidemiological importance).

### Ethics committee approval

This study involved the use of aggregate and publicly available data and was exempt from Institutional Review Board (IRB) approval.

## Results

### Calibration results

The final model produced results closely matching the four calibration targets: the number of persons presumed to have TB in 2021 (DHIMSII reported value 203,169; median simulated value 203,000, 95% uncertainty range [UR]: 183,000 – 224,000); the number of TB tests performed (DHIMSII reported value 150,782; median simulated value 151,000 [95%UR: 136,000 – 165,000]); the number of TB diagnoses (DHIMSII reported value 11,504; median simulated value 12,000 [95%UR: 10,000 – 13,000]); and proportion of pulmonary TB notifications that were clinically diagnosed (DHIMSII reported value 0.30; median simulated value 0.33 [95%UR: 0.22 – 0.46]) (**Fig D in** **S1 File**).

### Projected outcomes: Baseline scenario

**Fig 2:**
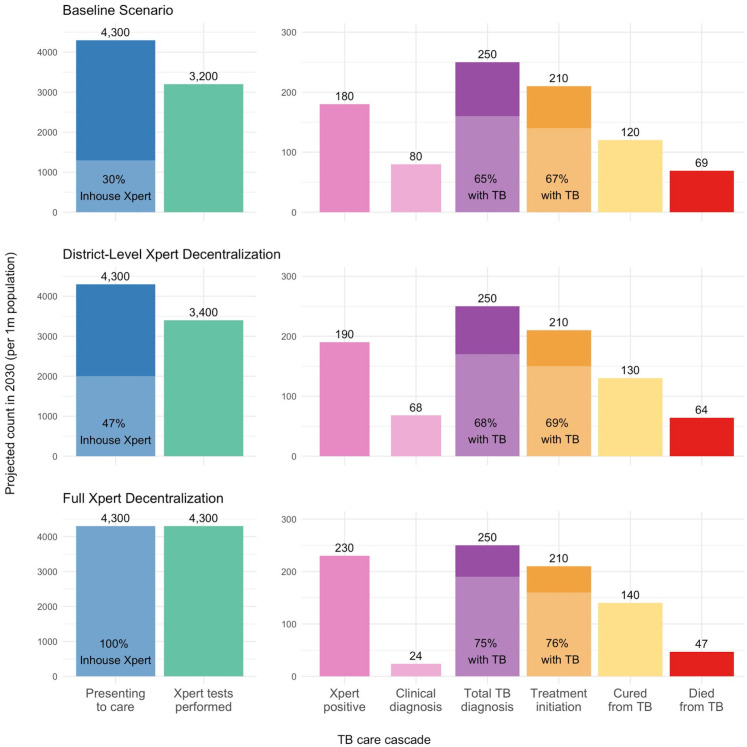
Projected TB Cascade of Care in Ghana in 2030. Panels show the projected steps of the TB care cascade in Ghana in 2030 under three scenarios as described in the main text: baseline (top), district-level Xpert decentralization (middle), and full Xpert decentralization (bottom). The bars represent the number of patients presenting to care (blue, with light shading showing the percentage of patients presenting at facilities with on-site Xpert), total Xpert tests performed (green), positive Xpert results and clinical diagnoses (pink), total TB diagnoses (purple, with light shading showing the proportion of people with true TB disease), TB treatment initiations (orange, with light shading showing the proportion of people with true TB), followed by the number of cured cases (yellow) and TB-related deaths (red). Median values per 1 million population in 2030 are displayed on top of each bar.

Based on the reported decline in TB incidence in Ghana [25], our model projected a 1.5% annual reduction in the number of patients seeking TB care, from a median of 5,500 [95%UR: 5,000 – 6,100] individuals per 1 million population in 2023 to a median of 4,300 [95%UR: 3,900 – 4,700] individuals per million population in 2030. The number of people with true underlying TB was also projected to decline, from 310 [95%UR: 230 – 410] to 240 [95%UR: 180 – 320] per million population from 2023 to 2030. Among these individuals, 70% were projected to present at facilities without current on-site Xpert capacity. If sputum testing for these facilities were done only at a higher tier laboratory, 38% [95%UR: 23 – 51%] of these patients, representing 1,100 [95%UR: 640 – 1,600] individuals with TB symptoms per million population, were projected to remain untested (**Fig 2**, top row).

The model projected a median of 3,200 [95%UR: 2,900 – 3,500] Xpert tests performed per million population in 2030, with 6% [95%UR: 4 – 7%] of tests being positive for TB. The model also projected a median of 250 [95%UR: 220 – 270] TB diagnoses per million population, with 33% [95%UR: 22 – 46%] of these diagnoses made clinically (i.e., without a positive Xpert confirmation). Among new TB diagnoses, an estimated median of 35% [95%UR: 17 – 50%] were false positives. In 2030, this translated to a median of 160 [95%UR: 120 – 210] individuals per million population being correctly diagnosed with TB, 85 [95%UR: 41 – 130] individuals receiving a false-positive diagnosis, and 74 [95%UR: 44 – 130] with TB infection going undiagnosed.

Among individuals diagnosed correctly with TB, a projected 85% [95%UR: 79 – 89%], or 140 [95%UR: 110 – 180] per million population, were projected to initiate treatment in 2030. Of these, our model estimated that 85% [95%UR: 64 – 97%] would be successfully treated, corresponding to 120 [95%UR: 84 – 160] people cured of TB disease per million population. Our model projected that 69 [95%UR: 36 – 120] per million population would die due to TB in 2030, including 29 [95%UR: 13 – 61] who remained untested, 23 [95%UR: 12 – 40] who received results but did not initiate treatment, and 14 [95%UR: 3 – 38] who died during treatment or retreatment.

### Projected impact of district-level Xpert decentralization

**Fig 3:**
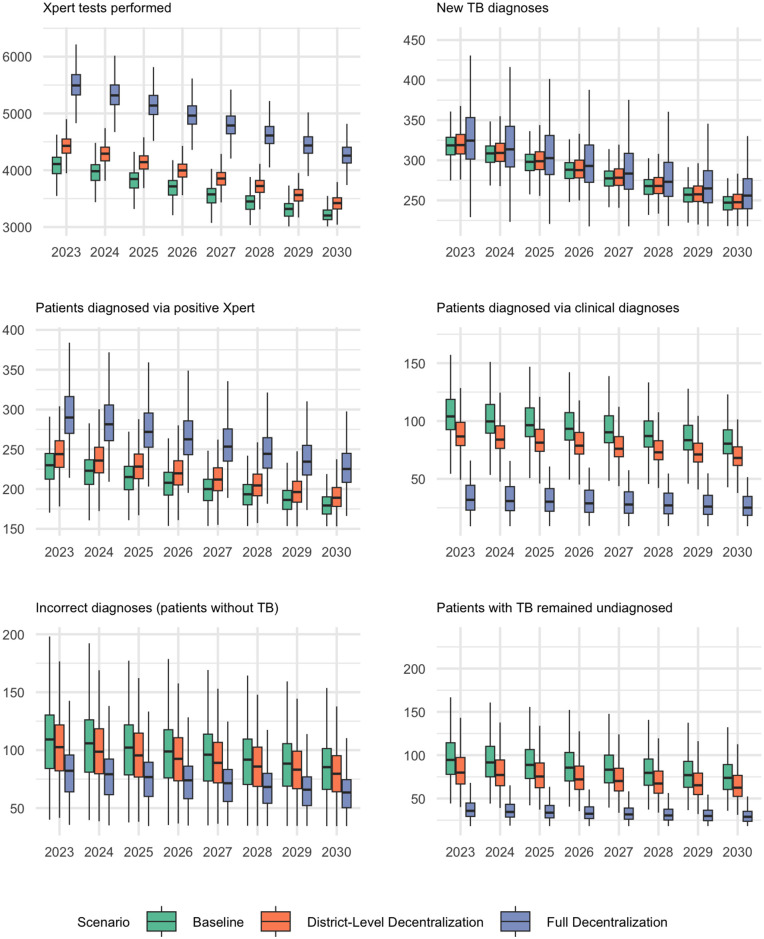
Projected Trends in TB Diagnostic Cascade in Ghana, 2023 – 2030. The panels illustrate the projected annual outcomes per 1 million population under three different Xpert placement scenarios (as described in the text) from 2023 to 2030. The boxes represent the interquartile range (IQR) for each outcome, while the whiskers extend to 95% uncertainty ranges, based on 1,000 calibrated simulations for each scenario. The focus is on observable measurements within the TB care cascade.

Providing Xpert testing at all district hospitals (addition of 96 Xpert instruments) resulted in a projected 24% reduction in the number of samples that had to be sent to higher tier facilities from 2023 to 2030 (**Fig 3**). This corresponded to an average of 800 [95%UR: 720 – 880] additional specimens being tested on-site per year, resulting in 300 [95%UR: 170 – 440] additional Xpert tests performed (representing testing for patients that would otherwise not have been performed), per million population in 2030, an 8% [95%UR: 4 – 13%] increase in overall testing volume (**Table E in** **S1 File**). These additional tests resulted in a projected 13 [95%UR: 7 – 23] per million population per year additional Xpert-confirmed diagnoses, with a corresponding decline of 15 [95%UR: 6 – 27] clinical diagnoses, per million population per year (2023–2030). Additional availability of Xpert in this scenario was projected to yield modest improvements in the accuracy of patient diagnoses, reducing the false-positive percentage from 35% to 32% and averting an average of 6 [95%UR: 5 – 19] incorrect diagnoses (patients without TB) per 1 million people per year. After accounting for the specificity of Xpert and clinical diagnoses as well as all forms of failures to testing, treatment initiation and treatment completions, the model projected that an average of 5 [95%UR: 2 – 11] additional people with TB infection would be cured annually per million population, along with a similar reduction in TB-related deaths. Over the period from 2023 to 2030, this would lead to a total of 43 [95%UR: 16 – 86] additional TB cures per million population with district-level Xpert availability.

### Projected impact of full decentralization

**Fig 4:**
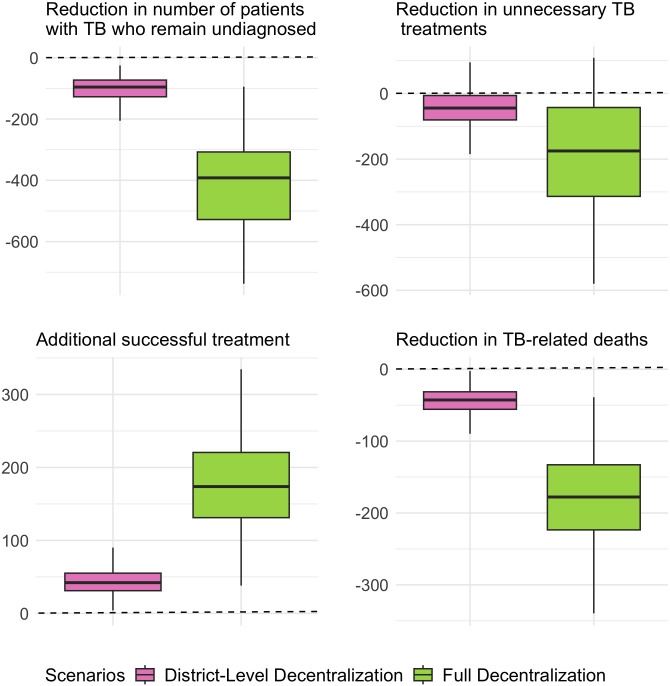
Projected Impact of Decentralized Xpert on TB Outcomes in Ghana over eight years. The panels illustrate the projected total differences in outcomes per 1 million population for selected clinical outcomes under district-level and fully decentralized Xpert scenarios compared to the baseline scenario, over an eight-year period. The boxes represent the interquartile range (IQR), while the whiskers extend to 95% uncertainty range, based on 1,000 calibrated simulations for each scenario.

Under a fully decentralized Xpert model, the number of Xpert tests performed in Ghana was projected to increase by 36% [95%UR: 19–54%] by 2030, leading to an average of 1,300 [95%UR: 730–1,800] additional tests per 1 million population per year compared to the baseline scenario (**Fig 3**).These additional tests resulted in a projected 56 [95%UR: 31 – 95] additional Xpert-confirmed diagnoses, with a corresponding decline of 63 [95%UR: 28 – 110] clinical diagnoses, per million population per year (2023–2030). Full Xpert decentralization was projected to reduce the false-positive patient diagnosis percentage to 25% [95%UR: 13 – 38%], averting an average of 22 [95%UR: 19 – 80] incorrect diagnoses (patients without TB) per 1 million people per year. Furthermore, the model projected that an average of 23 [95%UR: 10 – 46] more people with true TB disease would be cured annually per million population. This would also result in a similar reduction in TB-related deaths, with a projected 180 [95%UR: 77 – 370] fewer TB-related deaths per million from 2023-2030 (**Fig 4**).

### Sensitivity analysis

**Fig 5:**
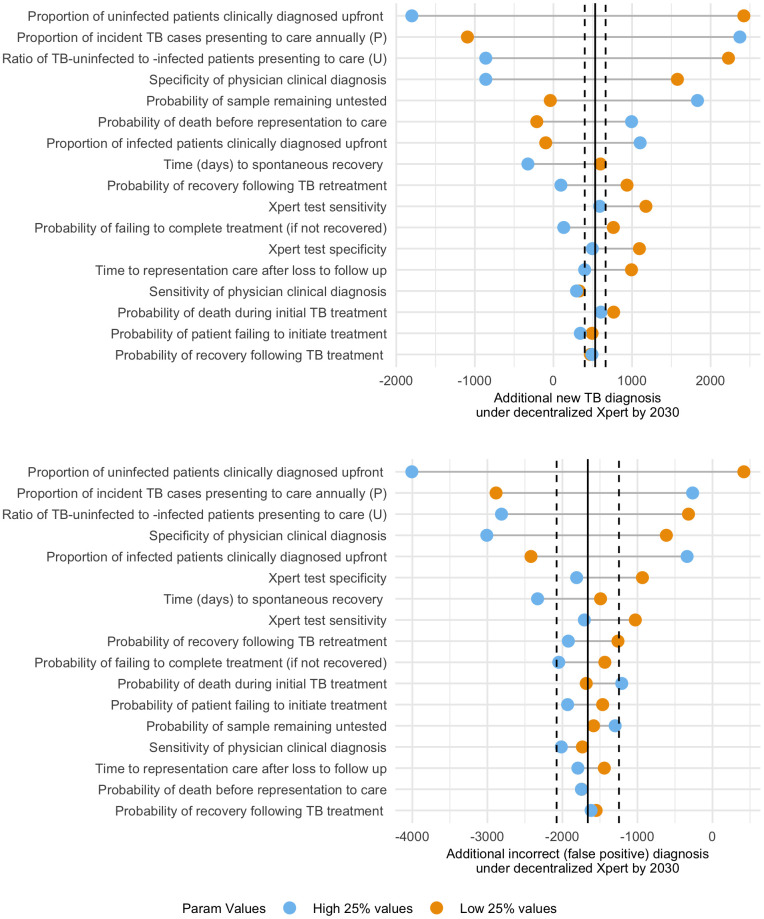
One-way sensitivity analysis of model parameters on the impact of district-level decentralized Xpert in Ghana. For those parameters most strongly associated with the primary outcome (absolute value of partial rank correlation coefficient, PRCC >0.1), the subset of simulations with that parameter in the highest quartile (in blue) was compared against simulations with that parameter in the lowest quartile (in orange). The primary outcome of interest was the change in the impact of district-level decentralized Xpert compared to the baseline scenario over an eight-year period for two results: additional new TB diagnoses (whether correct or incorrect) and reduction in incorrect (false-positive) diagnoses. Solid vertical lines mark the projected impact in the primary analysis, and dashed lines mark a threshold of +/-25% to identify differences of potential epidemiological importance.

In sensitivity analysis of the baseline model, the number of Xpert tests performed in 2030 was most sensitive to variations in the proportion of samples remaining untested, while the number of false-positive diagnoses was most sensitive to patient volume, probabilities of same-day clinical diagnosis, and Xpert test specificity (**Fig F in** **S1 File**). Our projections of impact from decentralized Xpert testing on total TB diagnoses and false-positive diagnoses varied most with patient volume, probabilities of same-day clinical diagnosis, specificity of clinical diagnosis, and proportion of samples remaining untested (**Fig 5**). Additional outcomes are presented in **Fig G in** **S1 File**.

## Discussion

This model of the TB diagnostic network in Ghana highlights both the potential value of decentralized molecular testing for TB and the importance of considering the full cascade of care when implementing novel diagnostic strategies. Specifically, both failure to perform testing (among sent-out samples) and continued reliance on clinical diagnosis play important roles in population-level outcomes. As such, strategies such as district-level decentralization – which only modestly reduces the number of specimens that can be tested on the same day – may have limited impact at the population level, whereas full decentralization, while having a much larger impact, requires substantial infrastructure and increases in testing volume. A major impact of decentralized molecular testing is in replacing clinical diagnoses with confirmed diagnoses; as such, the value of decentralized molecular testing may be more to increase confirmed TB diagnoses than to increase the number of total diagnoses made. These findings can be helpful to National TB Control Programs as they seek to utilize diagnostic resources most efficiently to reduce the population-level burden of TB at the country level.

Our findings align with previous studies, suggesting improved TB diagnostic accuracy and increased proportion of true TB cases among those diagnosed in the post-Xpert era [24,27–29]. Furthermore, it is important to assess Xpert diagnostic accuracy within the context of the underlying TB prevalence among individuals presenting with symptoms, as this can influence the positive predictive value [30,31]. For example, in our simulations, only 6% of people presenting with symptoms, and only 35% of people diagnosed clinically, were estimated to have underlying TB disease (**Table E in** **S1 File**). This results in an overall positive predictive value of 65% for new diagnoses. Expanding Xpert access could improve diagnostic accuracy, increasing the positive predictive value to 75% in a fully decentralized scenario through same-day, on-site testing, leading to more TB diagnoses and treatment initiation among individuals with underlying TB disease, while reducing false positive diagnoses and unnecessary treatment initiations. Achieving this, however, requires not only widespread implementation of testing infrastructure but also a considerable increase in testing volume (a 36% rise in testing volume in our fully decentralized scenario).

Our results highlight that shortening the diagnostic pathway by providing Xpert testing at peripheral health facilities has the potential to significantly improve case detection in Ghana. However, continued reliance on sputum samples can serve as a major challenge to TB diagnosis, as sputum can be difficult to collect, especially from children, severely ill patients and persons living with HIV. Novel diagnostic tools, such as urine-based lipoarabinomannan (LAM) tests and oral swab-based molecular assays offer promising alternatives to sputum-based tests for TB diagnosis [32]. These tests are non-invasive, easy to implement, and can specially expand testing access among individuals who are unable to produce sputum. However, there is often a trade-off between accessibility and diagnostic accuracy which can serve as a barrier to adoption and scale-up. A recent study in Ghana demonstrated the feasibility of using Determine TB LAM Ag assay for diagnosing TB among hospitalized HIV patients [33]. However, the study revealed significant gaps in the care cascade, with fewer than half of patients testing positive on the LAM assay initiating TB treatment. This was in part due to health care workers’ concern about the test’s specificity, which impacted clinical decision making. In response to these gaps, the Ghana Health Service has prioritized selected facilities – such as tertiary hospitals – to commence such point of care testing starting in 2025. These findings highlight the need to not only expand access to point-of-care TB diagnostics, but also to address the broader set of systematic barriers – including provider training, care coordination, and system capacity – to ensure that diagnostic gains effectively translate into timely TB treatment initiation and improved patient outcomes in Ghana.

These results also highlight why the observed impact of expanded Xpert testing on TB mortality has been limited at the country level [27,34–37]. First, expansion of Xpert testing to all sites where individuals present for diagnosis is challenging – and availability of Xpert testing at the district-level only modestly reduces proportion of samples remaining untested. Second, in the absence of on-site testing, a substantial number of people with TB are diagnosed clinically. While confirmation of TB diagnosis should be prioritized, it is also important to recognize the value of clinical judgment and early TB diagnosis when same-day molecular diagnosis is not available. Third, even with expanded molecular testing, a meaningful number of people are lost at each step of the cascade of diagnosis and treatment. Decentralized molecular testing closes one important gap in that cascade, but a diagnostic test alone cannot improve patient outcomes without strengthening the entire care cascade [34,38]. These findings illustrate the importance of thinking more holistically about the full cascade of diagnosis and treatment, from the patient perspective – including the value of decentralized molecular testing in addition to reducing failure to initiate treatment and complete treatment.

As with any simulation study, this analysis should be interpreted in light of several limitations. First, case notification numbers in the DHIMSII TB Screening Tool are approximately 30% lower than those reported by WHO (**Section D in** **S1 File**), and the locations or tiers at which underreporting occurs remain unclear. Facilities not reporting any data from 2019 to 2022 are excluded from our model. If underreporting is more pronounced at lower-level facilities, our model may underestimate the potential impact of decentralizing TB diagnostic services. Furthermore, even among reported cases, TB diagnoses at peripheral facilities may be under-represented if patients bypass these centers and seek care at higher-tier facilities or avoid seeking care altogether due to expected transportation costs and delays. In this case, the model may again underestimate the true value of decentralizing Xpert to peripheral levels. Second, we assumed full replacement of smear microscopy with Xpert for all TB diagnoses since 2020, in accordance with government policy; our model may therefore not fully reflect current diagnostic practices where smear microscopy is still used in smaller facilities. Third, our projected differences between decentralized scenarios and the baseline Xpert placement indicate substantial uncertainty (**Table E in** **S1 File**). These wide uncertainty bounds are driven by uncertainty in the scientific literature, calibration data, and differences from one simulation to the next. Importantly, our estimates of the impact of decentralized testing remain qualitatively similar across realistic simulations – as such, these results should be interpreted more as enhancing our understanding of likely effects than as providing precise estimates of impact under actual implementation. Fourth, our simulation simplifies the complexities of the Ghanaian TB diagnostic system by assuming universal sample transportation availability. Sputum specimens may not even be requested in settings where specimen transport is not available, though by calibrating to the DHIMSII TB Screening Tool, we should be capturing most causes for a test not being performed. Lastly, while decentralization was assumed to eliminate the probability of samples remaining untested in this model, equipment downtime, supply chain challenges, and other logistical barriers may still result in some missed tests [38]. Further loss can also occur after the test is performed, if the patient is not reachable by phone or other means. Our model excludes this intermediate loss between testing and successful notification. These nuances should be considered when interpreting our results and their broader applicability.

In summary, our findings underscore the importance of strategic policy planning for the expansion of Xpert testing in Ghana and other similar high-burden countries. While broad roll-out of Xpert to peripheral health facilities is promising, its feasibility remains constrained by practical challenges, including the cost of equipment and infrastructure, reliable supply chain, and limitations in staffing, training and maintenance at local health facilities [39]. As such, strategic and phased implementation (e.g., via the NoP network), paired with broader investments addressing systematic bottlenecks across the care cascade, may be more realistic than blanket decentralization in many settings.

In conclusion, our findings underscore that, while scaling up decentralized molecular testing like Xpert can have important epidemiological impact, decentralized testing may not be sufficient on its own to achieve high treatment initiation rates and reduce TB mortality. To achieve sustained improvement in TB control, such expansions must be accompanied by investment in system level bottlenecks across the care cascade, including patient referral mechanisms, provider training, reliable supply chains, and strong care coordination. Such a holistic approach will be critical to ensure that enhanced diagnostic capacity leads to sustained, long-term success in reducing TB burden.

## Supporting information

S1 File Supplementary information: Modeling framework, calibration details, and additional results. (DOCX)
