# Supplementary material for: Evaluating the impact of decentralized testing for tuberculosis in Ghana: A simulation model
Source: PLOS Glob Public Health. 2025 Nov 17;5(11):e0005302. doi: 10.1371/journal.pgph.0005302 (PMC12622812; doi:10.1371/journal.pgph.0005302)
Supplement: S1 File — (DOCX) [file pgph.0005302.s001.docx]

**S1 File. Supplementary Information: Modeling framework, calibration details, and additional results**

# Modeling Methodology

The District Health Information Management System (DHIMS-II) is a comprehensive health information system designed to capture, report, and analyze health data across Ghana's healthcare ecosystem (1). This web-based platform functions as a centralized tool for remotely collecting data from different tiers of the healthcare system. The system has been operational since 2012 and is available in all 260 health districts in Ghana. DHIMSII data from 2019 through 2022 is used to inform the simulation model structure.

## TB Diagnostic Network Representation

We represented the Ghana TB diagnostic network across seven tiers (**Figure A**). These tiers correspond to various levels within the Ghanaian healthcare system and are arranged based on their scope, ranging from national (tier 1) to local (tier 7) facilities. The first tier represents the “Ghana National Public Health Reference Laboratory”, followed by the “Zonal Public Health Reference Laboratory” in tier 2. These laboratories exclusively offer testing services without clinical capabilities. Tier 3 encompasses "Regional Hospitals" which offer both testing and clinical services. Tier 4 represents the “District Hospitals” in Ghana. Some “Regional Hospitals” or “Health Centers” may act as “District Laboratories”. Hospitals not classified as district hospitals are captured in tier 5. Tier 6 encompasses "Health Centers," and tier 7 encompasses "Clinics and Community Health and Planning Stations" (CHPS) as well as clinics. Facilities in tiers 4 to 7 provide clinical services (including diagnosis and treatment), with some offering in-house testing services contingent upon the availability of diagnostic equipment and personnel.


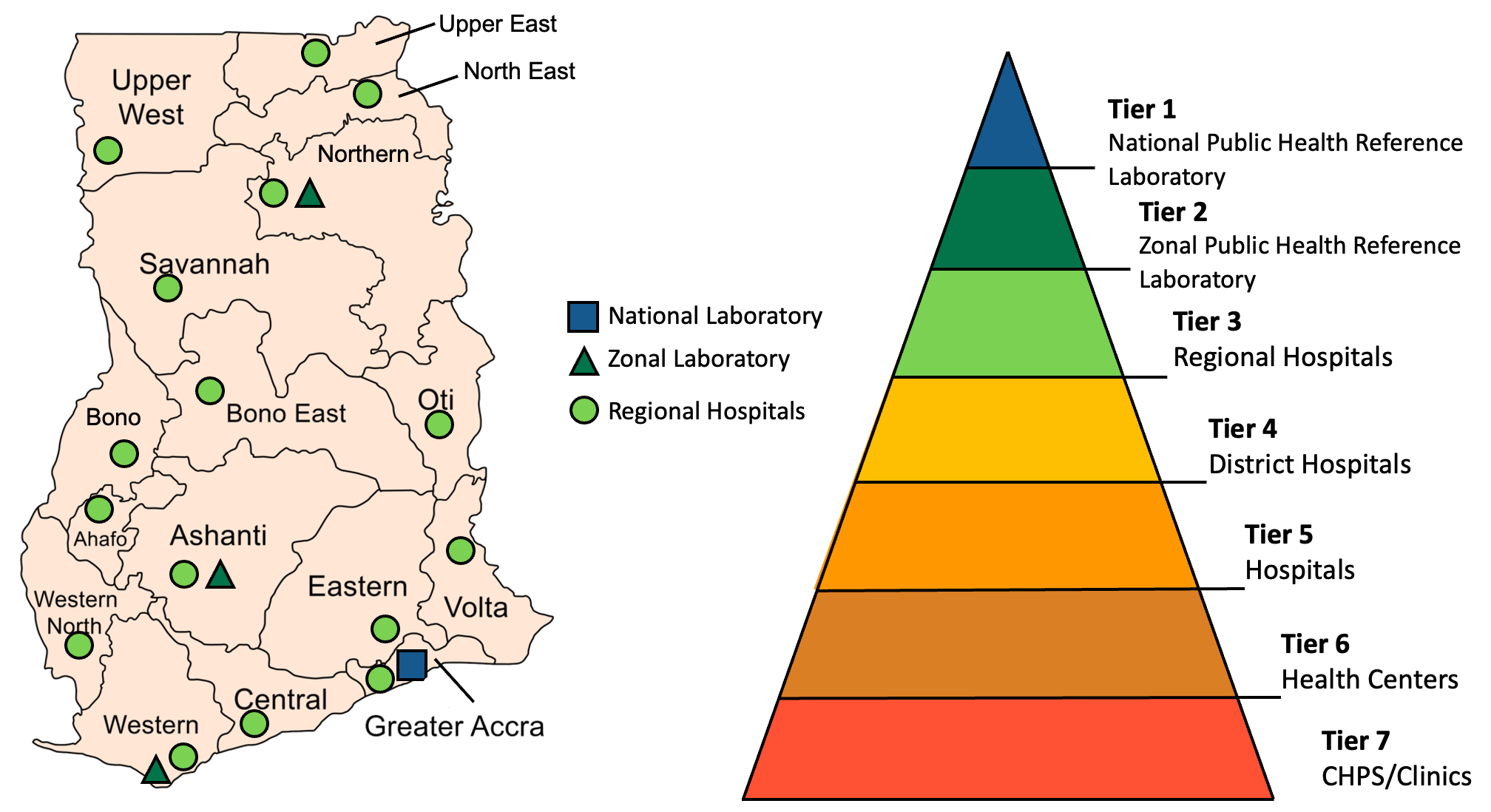


Figure A: The TB diagnostic network in Ghana is structured into seven tiers, categorized by their coverage from national (tier 1) to local (tier 7) levels. Each tier provides distinct services: national and zonal public health reference labs (tiers 1 & 2) offer testing exclusively, regional hospitals (tier 3) provide both testing and clinical services (e.g. diagnosis and treatment), while lower-level facilities (tiers 4 to 7) offer clinical services and perform laboratory testing when resources are provided. Base map from Wikimedia Commons: File: 2019 Regions of Ghana.png (<https://commons.wikimedia.org/w/index.php?title=File:2019_Regions_of_Ghana.png&oldid=808576493> [accessed Oct 27, 2025]), Licensed under Creative Commons Attribution 4.0 International license (https://creativecommons.org/licenses/by/4.0/deed.en).

## Modeling Scope

The Ghana diagnostic network encompasses over 9,500 facilities. While representing every single facility was outside the scope of our model, our aim was to capture all the necessary components to assess the impact of alternative TB diagnostics placements on patient- and population-level outcomes. Therefore, our model included facilities that reported an average of at least one TB case notification between 2019 and 2022 to DHIMSII (1), totaling 657 facilities. Additionally, in partnership with local collaborators, we integrated 27 district-level facilities essential for TB diagnosis, not listed in DHIMS-II. Furthermore, we included 9 Xpert site facilities from Ghana's Xpert facility list lacking TB notifications in DHIMS-II. Moreover, we incorporated 10 facilities identified in a Ghana Health Service Diagnostic Network Optimization study, even though they did not report notifications in DHIMSII (2). In total, our model comprises 703 health facilities, including 170 Xpert sites (**Table A**).

Table A: List of modeled facilities and Xpert availability

| Tier | Tier name | Number of facilities | Xpert Availability (Baseline) | Xpert Availability (District-level decentralization) |
| --- | --- | --- | --- | --- |
| 1 | National Public Health Reference Laboratory | 1 | 1 | 1 |
| 2 | Zonal Public Health Reference Laboratory | 1 | 1 | 1 |
| 3 | Regional Hospitals | 8 | 8 | 8 |
|  | *also acting as District Laboratories | 8 | 8 | 8 |
| 4 | District Hospitals | 218 | 122 | 218 |
| 5 | Hospitals | 92 | 21 | 21 |
|  | *acting as District Laboratories | 4 | 3 | 3 |
| 6 | Health Centers | 265 | 4 | 4 |
| 7 | CHPS/Clinics | 106 | 2 | 2 |
| Total |  | 703 | 170 | 266 |

## Facilities Population Catchment Area

We assumed that patients always present to the nearest facility with the shortest travel time for diagnostic and treatment services. Despite certain limitations, this simplifying assumption allowed us to map a one-on-one relationship between each geographical area with the simulated facilities in our model. Following this logic, each simulated facility was associated with a “population catchment area” representing the spatial distribution of people with the closest travel time to that facility (**Figure B**).

We used AccessMod (version 5.6.0) to estimate the population catchment areas for each modeled facility (3). Initially, we created terrain, population, and infrastructure (including roads) tables within ArcGIS Pro (version 3.2.0) (4), which were then projected and tailored to Ghana's geography. These tables were subsequently imported into AccessMod to calculate the shortest travel times to each facility. The resulting image files are utilized to delineate catchment zones for each facility. To represent varying travel times for patients within each catchment zone, we further divided each zone into ten concentric bands based on travel times.

Population estimates were based on the population distribution data from WorldPop for the year 2015.(5) AccessMod requires a population cluster file, where each cluster denotes the population within uniform distance clusters. However, such files are only available for 2015 and 2020. To bridge this gap, we estimated a smooth growth curve for Ghana's population from 2015 to 2020, with growth rates as follows: 2015 – 2.39%, 2016 – 2.37%, 2017 – 2.26%, 2018 – 2.15%, 2019 – 2.11%, and 2020 – 2.09%(6). The population map from 2015 serves as the base, and we apply the respective growth factors to estimate population sizes for each year from 2015 onward. This allowed us to break down the population map into 1 km clusters and map the proportion of the population falling within each concentric band, facilitating the calculation of distances from the facility for each new patient in the model.

**
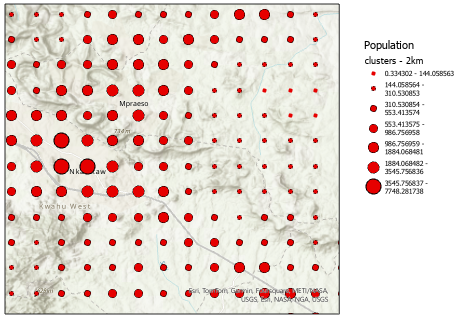

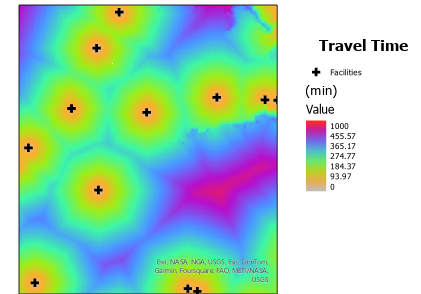
**

Figure B: Facilities Population Catchment Area. The left panel provides a depiction of the population breakdown in ArcGIS. It displays the Bono East region's Atebubu Amantin, Sene West, and Sene East districts, as well as the Sekyere Afram Plains North district in the Ashanti Region, divided into 2 km clusters. The size of the circles represents the population density in each cluster. The right panel illustrates catchment zones for selected facilities within this area, marked in black. Using travel time data from AccessMod, we determined the nearest facility for population residing in each location on the map and established catchment zones within colored concentric bands. The figures in the left and right panels were generated via ArcGIS Pro (version 3.2.0) and AccessMod (version 5.6.0) respectively. Base map was imported directly from Africa GeoPortal ([https://www.africageoportal.com](https://www.africageoportal.com/?utm_source=chatgpt.com)), a publicly available Esri-powered platform that provides access to geographic data from African countries and regions. The Africa Geoportal data are distributed under the Esri Master License Agreement (https://www.esri.com/content/dam/esrisites/en-us/media/legal/ma-translations/english.pdf).

### Estimating the population size of patients presenting to care with TB infection

The simulations ran from the year 2021 to year 2030. At the onset of each year, we simulated a population of new patients seeking care including individuals with TB infection and those without TB (but experiencing TB-like symptoms).

In the absence of a representative model of TB transmission dynamics in Ghana, we simplified our approach to estimating the population of patients with TB infection seeking care. We assumed that the number of individuals presenting to care who were truly infected with TB in each year was directly proportional to the number of new TB incident cases in that year. To project future TB incident rates in Ghana, we utilized the reported TB incidence estimates from the World Health Organization (WHO) and assume an annual reduction of 2.7% in incidence rates from 2017 to 2020 (7). This trend was extended forward to forecast the future TB incident rates to year 2030 as follows:

$$rInc\left( t \right)=rInc_{2020}\times\left( 1-0.027 \right)^{t-2020}$$

where *rInc_2020* represents TB incidence rate (per 100,000) in 2020 and *rInc(t)* is the projected TB incidence rate in year *t* (*t* between 2021 to 2030)(**Table B**).

Following this, we introduced a simulation parameter, denoted as the proportion of new TB incident cases that seek care annually (P). We estimated the prior distribution for this parameter based on the WHO's reported ratio of TB treatment coverage in 2021, calculated as the number notified divided by the estimated incidence (7). Finally, we estimated the number of patients presenting to each facility who are truly infected with TB in each year as follows:

$$n\_Inf(i,t)=pop\left( i,t \right)\times P\times{rInc\left( t \right)}/{100,000}$$

$$n\_inf(t)=\sum_{i=1}^{N} n\_inf\left( i,t \right)$$

where *n_inf(i,t)* represents the number of patients presenting to facility *i* who are truly infected with TB in year *t*, *pop(i,t)* represents the population catchment size for facility *i* in year *t*, *P* is the proportion of incident TB cases presenting to care, and *rInc(t)* is the projected TB incidence rate (per 100,000) in year *t* (*t* between 2021 to 2030). Furthermore, *n_inf(t)* represents the total number of patients with TB presenting to care in year *t* at the national model, *i* represents simulated facilities in the model (*i* = 1,…,*N*), and *N* is the total number of facilities in the model (*N* = 703). When P=1, *n_inf* corresponds to the number of incident TB cases in each year (**Table B**).

Table B: Estimated population size and TB incident cases in Ghana, 2020-2030.

| Year | Population size | TB incidence rate (per 100,000 persons) | Estimated TB incident cases |
| --- | --- | --- | --- |
| 2020 | 32,180,401 | 140 | 45,053 |
| 2021 | 32,833,031 | 136.2 | 44,725 |
| 2022 | 33,475,870 | 132.5 | 44,370 |
| 2023 | 34,121,985 | 129.0 | 44,005 |
| 2024 | 34,777,522 | 125.5 | 43,639 |
| 2025 | 35,439,785 | 122.1 | 43,270 |
| 2026 | 36,102,736 | 118.8 | 42,889 |
| 2027 | 36,767,488 | 115.6 | 42,499 |
| 2028 | 37,435,099 | 112.5 | 42,103 |
| 2029 | 38,104,805 | 109.4 | 41,699 |
| 2030 | 38,775,850 | 106.5 | 41,287 |

Of note, while our spatial model (AccessMod) relies on WorldPop 2015 data to estimate population distribution and facility catchment areas, the WorldPop data ends in 2020, and it does not provide official population projections beyond that point. To address this limitation, we used the Macrotrends population projections for Ghana from 2020-2030 to inform the population of TB infected cases in the model (8).

### Estimating the population size of all patients presenting to care

In addition to patients presenting to care who are truly infected with TB, the simulated population also includes individuals presenting to care with TB-like symptoms who are not infected with TB. We establish the size of this population to be proportionate to the number of patients with TB infection presenting at each facility. Following this, we model the total population of new patients presenting to care as follows:

$$n(i,t)=n\_inf(i,t) \times\left( 1+U \right)$$

$$n(t)=\sum_{i=1}^{N} n(i,t)$$

where *n(i,t)* represents the number of new patients presenting to facility *i* (*i*=1,…,*N*) in year *t* (*t=* 2021,…,2030), *n_inf(i,t)* is the number of TB-infected patients presenting to facility *i* in year *t*, *U* is the number of persons without TB presenting to care, per person with TB, and *N* is the total number of facilities in the model (*N* = 703). Furthermore, n(t) represents the total population presenting to care in year *t* at a national level.

## Simulated Patient Pathway

The model represents a patient’s pathway through the TB diagnostic and treatment cascade (**Fig 1** in the main text). The simulation begins with the patient’s arrival at a health facility. A sputum sample is taken from each patient upon arrival. If TB testing is available at the facility, the sample is tested ‘*in house’* and the patient receives their diagnosis on the same day. Alternatively, if Xpert testing is unavailable at the facility, the sample is transported to the nearest facility with testing capacity (‘*sample sent out’*). In this scenario, the patient is discharged and awaits the diagnosis result via phone.

To account for scenarios where a symptomatic patient presents at a facility without in-house testing and is treated based on clinical judgment, we include a pathway for a proportion of patients arriving at facilities without Xpert. These patients will receive a TB diagnosis based solely on clinical assessment (‘*upfront TB clinical diagnosis’*). Although sputum samples will still be collected and sent for testing, the results will not affect the treatment trajectory.

Sputum samples are transported to other facilities via ground transportation. Samples sent out to other facilities may get lost during the transport or the testing process may be unsuccessful for any number of reasons. These instances in sum are considered ‘*probability of samples remaining untested’* and modeled as a single parameter that we estimate from the DHIMSII fields of the number of TB patients tested vs the number of TB patients presumed. We assume no sample remain untested when Xpert testing is available onsite.

Once sputum samples are tested, results are returned to the original facility for review by a physician. The physician then shares the results and diagnosis with the patient. Physicians have the option to override negative test results and make a clinical diagnosis for patients believed to have TB infection despite testing negative for TB. Patients diagnosed with TB are prescribed a 6-month first-line TB treatment regimen, which they may receive at the closest facility with treatment resources available. However, a proportion of patients will not initiate TB treatment and are considered ‘*failure to initiate treatment’*.

Most patients undergoing TB treatment recover from the disease. However, a subset of patients face the risk of *‘incomplete treatment’*, death during treatment, or treatment failure. Upon completion of treatment, some patients do not resolve their symptoms and remain infectious for TB. They will return to care immediately and initiate a new course of retreatment, which may result in full recovery or death. Patients who die during TB treatment (or retreatment) are assumed to remain infectious throughout the treatment period and exit the model upon death. Individuals without TB infection are not affected by the TB treatment.

Following this logic, our model captures three pathways leading to failed treatment among TB infected patients (blue box-**Fig 1**). All of these instances are managed similarly. Patients either die, re-present to care, or in rare instances, spontaneously recover from the infection. We assume that individuals with untreated TB exhibit a fixed probability of re-presentation to care each month, generating the time to re-presentation to care from a corresponding geometric distribution. Additionally, we estimate the probability of case fatality among TB patients who remain untreated and apply this at the time of re-presentation to care to determine if the patient dies at that time or successfully re-presents to care. Persons surviving with untreated TB will undergo spontaneous recovery from infection after 10 years.

## Additional assumptions regarding travel times, resource availability and turn-around-time

Travel times for patients traveling to the presenting facility are based on calculated travel times output from Accessmod.(3) Distance is determined by geographic barriers (water, mountains, etc.) and available roads. Accessmod uses the type of terrain and average walking, bicycling and car travel speeds to determine the time it takes to travel any distance from a patient’s home to the presenting facility. Travel times between facilities are based on distance along roads between the presenting and testing facility with an average travel time per kilometer based on road quality.

In accordance with current practice in Ghana, the model assumes phone communication of results to patients, improving the effective turnaround time of testing in comparison to the practice of patients returning to the presenting facility to receive results. We exclude potential loss to follow up among patients with test results but who could not be reached to communicate a final diagnosis, as such data are not available via DHIMS-II. Treatment is then initiated at the nearest medical facility to the patient’s home.

# Calibration Methodology & Results

The baseline scenario simulates TB testing using 170 Xpert machines, which closely approximates the current TB diagnostic network in Ghana (**Table A**). Patients with symptoms indicating possible TB infection present to the closest facility, and if Xpert is available onsite, testing is carried in-house. If Xpert is not available on site, the sample is sent out to the closest facility equipped with Xpert testing. This scenario serves as the basis for calibrating the baseline model.

The calibration methodology involves iteratively adjusting the model's input parameters to minimize discrepancies between simulated results and observed TB outcomes in Ghana. To achieve this, we first estimate a set of prior distributions for all simulation parameters based on existing data. Next, we identify and estimate key calibration targets, which represent important aspects of the TB care cascade in the country. We then apply a Bayesian Sampling-Importance-Resampling (SIR) framework to calibrate the model resulting in an updated set of parameter distributions.

## Prior Distributions

The model includes 18 parameters that describe various aspects of population presentation, TB diagnostics, and outcomes for patients seeking care. The exact values of these parameters are unknown, but we leverage existing data from DHIMSII and the literature to derive prior distributions, which serve as the best estimates for these parameters throughout the calibration process (**Table C**).

Table C: List of model parameters, prior distributions and descriptions.

| Parameter | Prior Distribution^1^  (mean) [Range] | Description/Reference |
| --- | --- | --- |
| Proportion of incident TB cases presenting to care annually (P) | Uniform [0.15 – 0.35] | TB treatment coverage (notified/estimated incidence), 2022 (9) |
| Number of persons without TB presenting to care, per person with TB (*U*) | Uniform [14 – 30] | The estimated ratio of those presumed for TB to those diagnosed with TB in DHIMSII (2021-22)(1). The range is then determined empirically to address underlying uncertainty. |
| Proportion of infected patients clinically diagnosed with TB upfront in the absence of Xpert | Uniform [0 – 0.3] | (10) |
| Proportion of uninfected patients clinically diagnosed with TB upfront in the absence of Xpert | Uniform [0 – 0.05] | Assumption |
| Probability of sample remaining untested | Uniform [0 – 1] | The estimated ratio of individuals tested for TB to those presumed with TB in DHIMSII (2021-22)(1). The range was then determined empirically to address underlying uncertainty. |
| Probability of patient failing to initiate treatment | Beta (0.14) [0.1 – 0.4] | Estimated ratio of individuals diagnosed with TB who did not start treatment in DHIMSII (2018 – 2020).(1) The range is estimated based on literature. (11) |
| Xpert test sensitivity | Beta (0.85) [0.78 – 0.92] | (12) |
| Xpert test specificity | Beta (0.99) [0.98 – 1] | (12) |
| Sensitivity of physician’s clinical diagnosis among those with TB who tested negative for TB | Uniform [0 – 0.3] | (10) |
| Specificity of physician’s clinical diagnosis among those without TB who tested negative for TB | Uniform [0.99 – 0.999] | Assumption  In our model, we assumed a very high specificity of clinical diagnosis for patients with a negative Xpert result. This assumption was necessary because we have simplified the clinical evaluation to apply uniformly to all negative results, regardless of symptom presentation (i.e. severity of illness). While clinicians in real-world settings consider numerous signs and symptoms before making a diagnosis, we chose not to model those complexities. Moreover, due to the high number of uninfected patients presenting for care, the abundance of negative results necessitated a high specificity to avoid unrealistically high false-positive diagnoses |
| Probability of recovery following TB treatment ^2^ | Beta 0.85 [0.63 – 1] | The proportion of bacteriologically confirmed and clinically diagnosed new and relapsed patients reported as ‘cured’ during/post treatment in the DHIMSII treatment dataset (2018-2020).(1) The range is determined empirically to address underlying uncertainty. |
| Probability of incomplete treatment among patients who do not recover after receiving TB treatment | Beta 0.33 [0.2 – 0.46] | We calculated the probability of incomplete treatment specifically among patients who did not recover after receiving TB treatment. This was done by dividing the number of bacteriologically confirmed and clinically diagnosed new and relapsed patients reported as 'lost to follow-up' (incomplete treatment outcome) during/post treatment by the number of patients who were not reported as ‘cured’ during/post treatment in the DHIMSII treatment dataset (2018-2020).(1) |
| Probability of death during initial TB treatment among patients who don't recover after receiving TB treatment and do not complete treatment | Beta 0.9 [0.5 – 1.0] | We calculated the probability of deaths specifically among patients who do not recover after receiving TB treatment and do not complete treatment.  This was done by dividing the number of bacteriologically confirmed and clinically diagnosed new and relapsed patients reported as ‘dead’ during/post treatment by the total number of patients who were not reported as ‘cured’ or 'lost to follow-up' (incomplete treatment outcome) during/post treatment in the DHIMSII treatment dataset (2018-2020).(1) The range is determined empirically to address underlying uncertainty. |
| Probability of recovery following TB retreatment^3^ | Beta 0.85 [0.63 – 1] | The proportion of bacteriologically confirmed and clinically diagnosed new and relapse patients reported as ‘cured’ during/post treatment in the DHIMSII treatment dataset (2018-2020)(1). The range is determined empirically to address underlying uncertainty. |
| Time to death during TB treatment/retreatment experiencing this outcome | Uniform [0 – 180] days | Assuming a TB treatment period of 6 months. |
| Time (months) to re-presentation to care after^4^ | Beta 0.1 [0.05 – 0.15] | We assume that individuals with untreated TB experience a fixed probability of re-presentation to care in each month and generate the time to representation to care from a corresponding geometric distribution. In the absence of a specific reference, we estimated this mean value using empirical data and expert opinion. |
| Probability of death before re-presentation to care | Uniform [0.3 – 0.8] | Assuming a TB case-fatality rate of 35% in 2022 (9), we first estimated the total number of deaths among incident TB cases to be 15,400 (35% of 44,000 = 15,400). Then, using a notification rate of 37%, we estimated the number of individuals notified and treated for TB to be 16,280 (37% of 44,000 = 16,280). Among those treated, the mortality ratio reported in DHIMSII was 10%, resulting in an estimated 1,628 deaths. Subsequently, it could be assumed that the remaining mortality (13,772 deaths) occurred among individuals who sought care at some point and were not notified or treated successfully (28,600 cases). This would yield a case-fatality rate of 48% regardless of the duration until re-presentation. The range is determined empirically to address underlying uncertainty. |
| Time (days) to spontaneous recovery | Uniform [1 – 3600] | Assumption |

1 Prior distributions are represented as the median (2·5-97·5 percentile) for Beta distribution, and as [minimum – maximum] for uniformly distributed priors. Uniform priors are chosen to be minimally informative, and their ranges were adjusted empirically such that well-fitting models had values far from the endpoints.

2 Initial TB treatment can result in four outcomes including recovery, incomplete treatment, death or relapse (**Fig 1**). To allow independent sampling for simulation parameters, we model the probability of recovery among all patients receiving treatment, the probability of incomplete treatment among who do not recover, and the probability of death during treatment among those who do not recover and do not complete treatment. The remaining patients will relapse to active disease and will receive TB retreatment.

3 TB retreatment can result in two outcomes including recovery or death. We model the probability of recovery among all patients receiving retreatment, the remaining patients will die during retreatment.

4 Patients living with untreated TB may re-present to care, die from untreated TB or spontaneously recover. We assume that individuals with untreated TB experience a fixed probability of re-presentation to care in each month and generate the time to re-presentation to care from a corresponding geometric distribution. Those with untreated TB also experience a fixed probability of death before re-presentation. Individuals who survive with untreated TB will spontaneously recover after 10 years.

## Calibration Targets

We focus on four primary calibration targets of interest, derived from DHIMSII data, to represent key aspects of TB care cascade in Ghana:

- **Number of persons presumed to have TB:** The number of simulated patients presenting for care in our model (each with symptoms meeting criteria for presumed TB).
- **Number of TB tests performed:** The number of Xpert tests performed in the model.
- **Number of TB diagnoses:** These are the number of patients diagnosed with TB (the final diagnosis of the patient irrespective of TB test results) and thus constituting the number of TB notifications.
- **Proportion of TB diagnoses that are clinically diagnosed:** This is the proportion of all diagnoses of TB for which there is not a positive TB test (a diagnosis without laboratory confirmation).

Our primary data source to estimate the calibration targets was DHIMS-II, available for the years 2019 to 2022. However, due to changes in the TB diagnostic network structure during this period and the impact of COVID-19, we focused on calibrating the model to estimates from 2021 and 2022 and applied them to calibrate simulated projections in year 2021 (**Table D**).

Table D: Calibration targets and description

| Calibration targets  (for year 2021 in the model) | Target Value [Range] | Description/Source |
| --- | --- | --- |
| Number of persons presenting to care | 203,169 [182,852 – 223,486] | Average DHIMSII reported number of patients presumed with TB in 2021-2022, +/- 10% uncertainty range (1) |
| Number of Xpert tests performed | 150,783 [135,704 – 165,861] | Average DHIMSII reported number of TB test performed in 2021-2022, +/- 10% uncertainty range (1) |
| Number of TB notifications | 11,504 [10,354 – 12,654] | Average DHIMSII reported number of TB notifications in 2021-2022, +/- 10% uncertainty range (1) |
| Proportion clinically diagnosed | 30% [27% – 33%] | Average DHIMSII reported proportion of pulmonary TB notifications that are clinically diagnosed in 2021-2022,  +/- 10% uncertainty range (1) |

## Calibration Methods and Results

We used a Sampling-Importance-Resampling (SIR) framework to calibrate the model. This framework allowed us to update the parameter estimates by sampling from the prior distributions and resampling based on how well the simulated outcomes aligned with the calibration targets. This iterative process refined the model's parameter values, enhancing the accuracy and reliability of the simulated TB projections.

The model includes 18 input parameters, listed in **Table C**. Each parameter was characterized by a prior distribution representing our best knowledge of its potential value based on literature and prior studies. For some model parameters where no information was available, we used a uniform distribution with an empirical range and manually revised this range to achieve acceptable coverage in the posterior distribution. Following this, we ran a large batch of simulations with 50,000 models by randomly sampling prior distributions for each parameter and running simulations to the year 2021 (the calibration year). **Figure C** illustrates the distribution of simulated outcomes corresponding to each primary target across the initial 50,000 simulations before calibration.


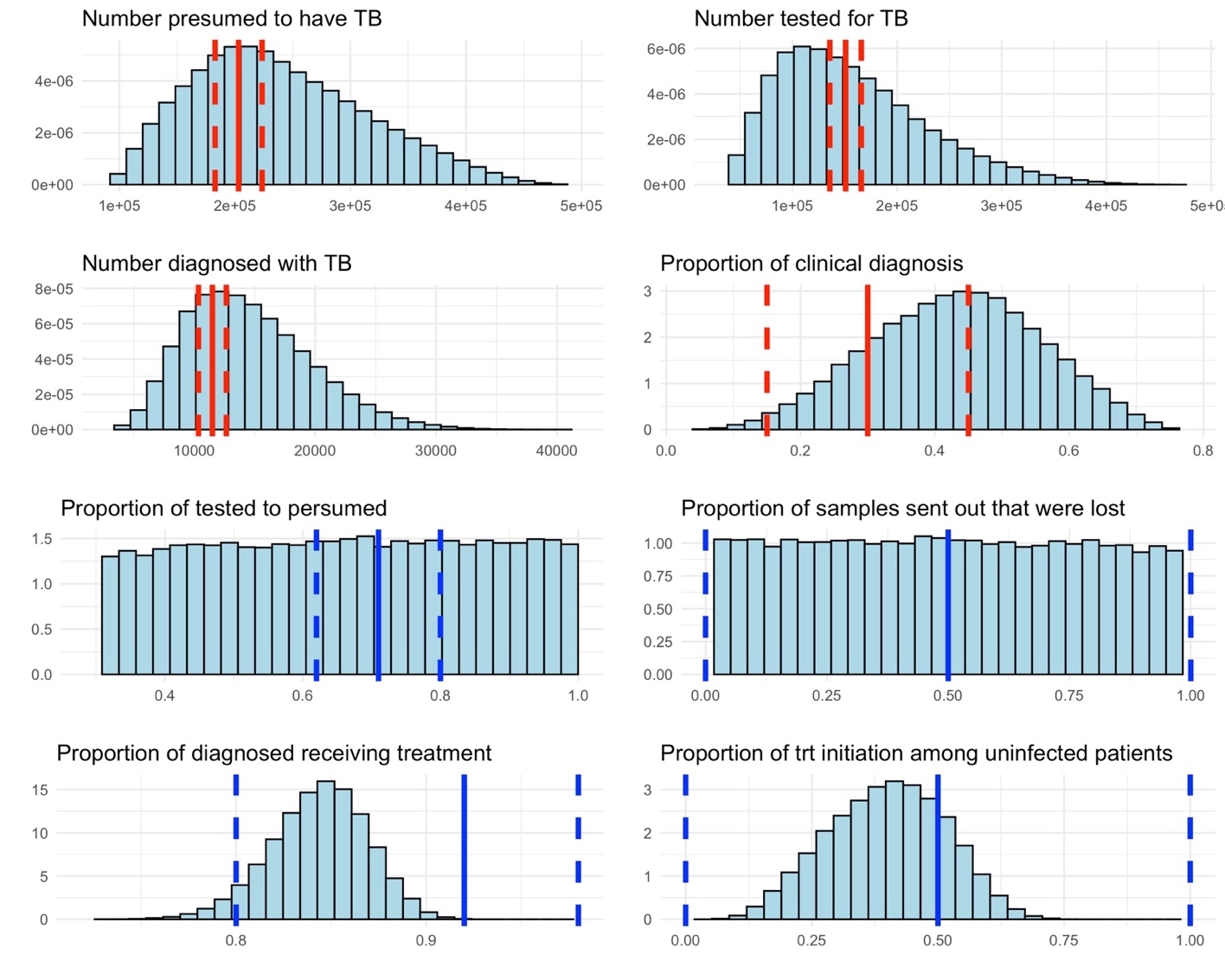


Figure C: Distribution of simulated outcomes corresponding to each primary target across the initial 50,000 simulations before calibration. The x-axis corresponds to the target variables, while the y-axis represents the proportion of simulations within each bin.

In the next step, we assessed the goodness of fit using a *log-likelihood function*, applying a logit-normal distribution for targets spanning between 0-1 and a log-normal distribution for others. Using the estimated log-likelihood as weights, we resampled 1,000 parameter sets that represented a collection of best-fitting simulations. This resampled set included 386 unique parameter sets, accommodating instances where certain parameter sets may be duplicated due to their higher weight, indicative of a superior calibration fit. **Figure D** illustrates the distribution of simulated outcomes corresponding to each primary target across the resampled 1,000 simulations, indicating a close fit to all primary targets in year 2021.


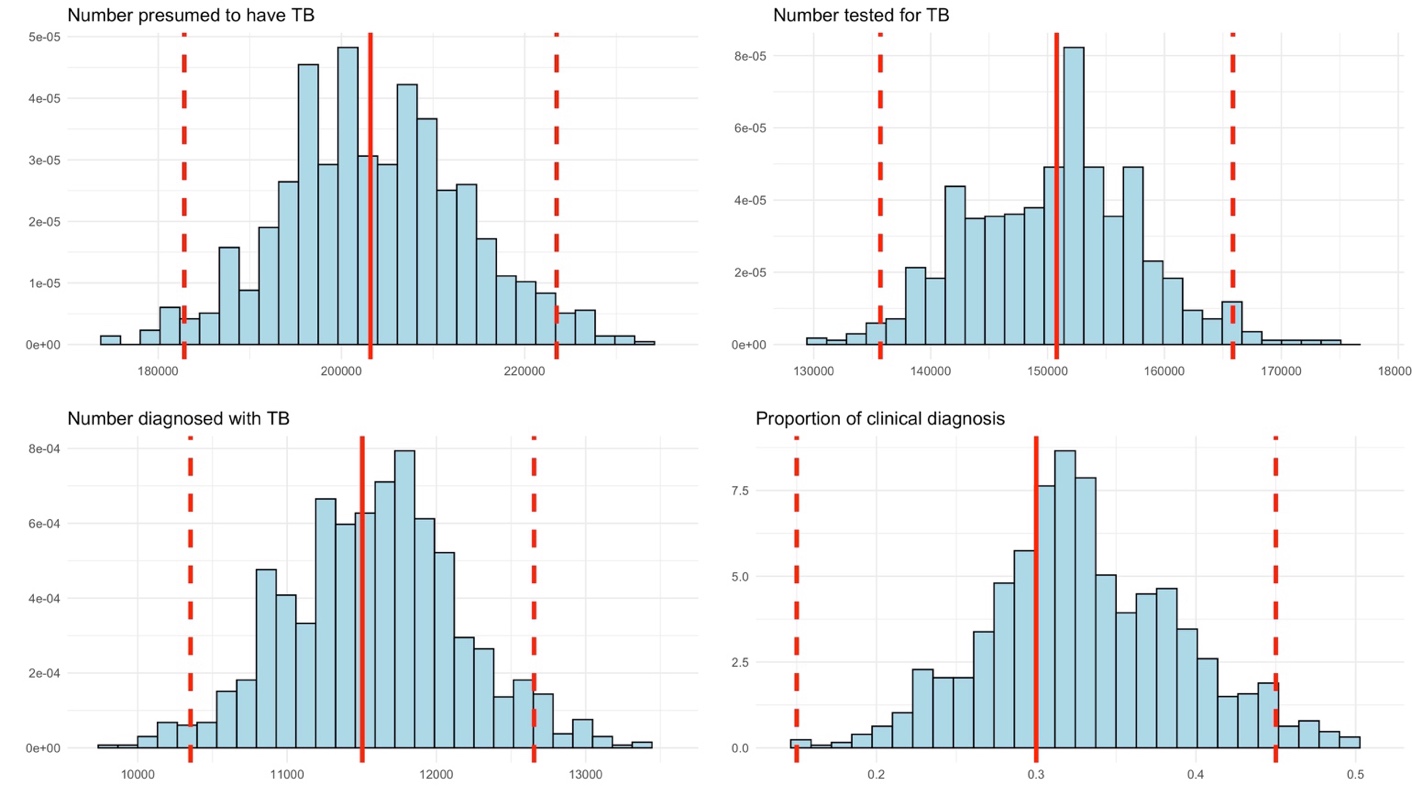


Figure D: Calibration fit to primary targets. The x-axis represents the target variables, while the y-axis indicates the proportion of simulations within each bin. Red bars correspond to the calibration targets for the year 2021, with the solid line indicating the median and the dashed line showing the uncertainty range from Table D. Blue bars show the projected outcome distribution from the 1,000 resampled models for the year 2021.

The comparison between prior and posterior distributions for model parameters is shown in **Figure E.** The prior distributions (shown in red), showcase our initial understanding or assumptions about the parameter values. In contrast, the posterior distributions (shown in green) reflect the updated distributions after incorporating information from the calibration process. This comparison allowed us to visualize how beliefs about parameter values have evolved based on observed data and model fitting. We used these 1,000 resampled parameter sets in all subsequent simulations of different diagnostic scenarios.


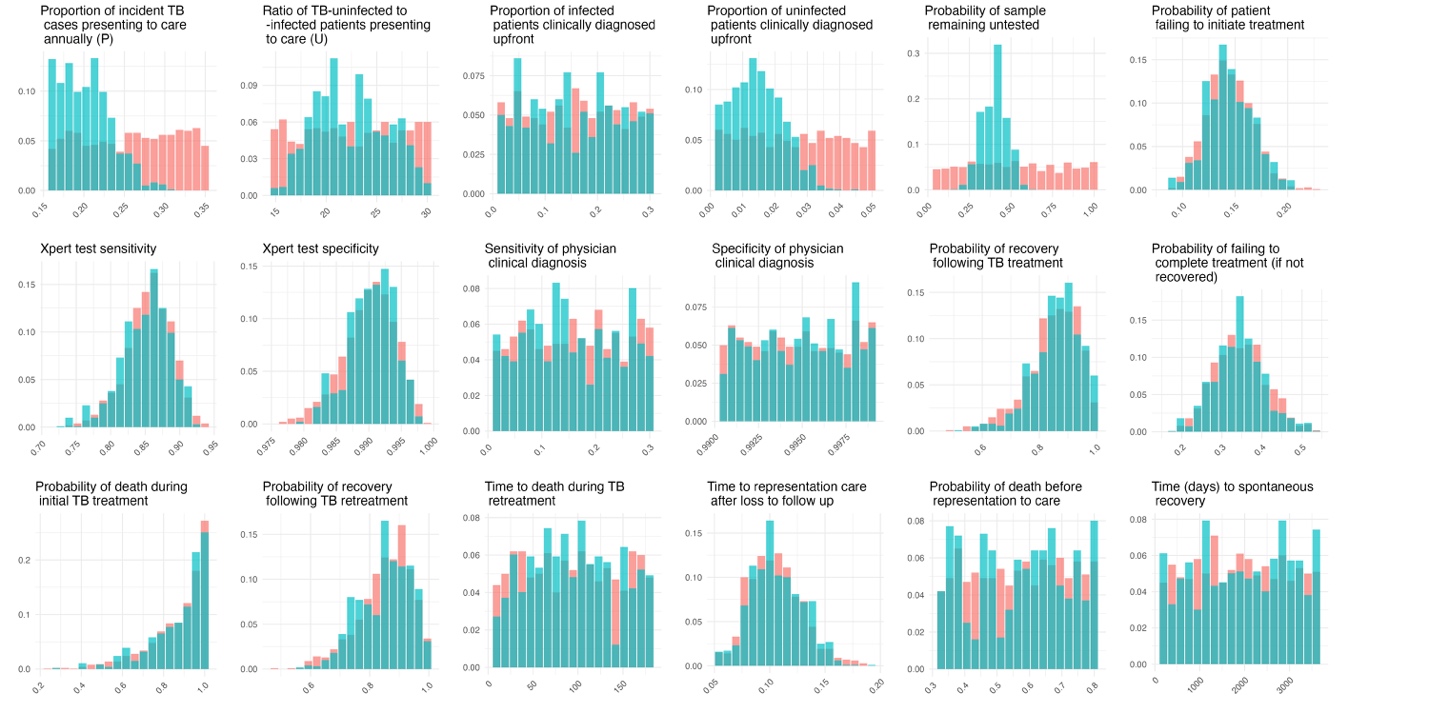


Figure E: Comparison of Prior and Posterior Distributions for Model Parameters. The x-axis denotes the range of each parameter, contrasting the prior distribution (in red) with the posterior distribution (in green).

## Experimental Scenarios

The primary simulation scenarios, aimed at representing large-scale configurations, present distinct approaches to the placement of Xpert testing within healthcare systems as follow:

- **Current Placement of Xpert (Baseline)**: Reflecting the existing TB diagnostic network in Ghana as of 2023, this scenario places Xpert testing at 170 facilities (**Table A**). In the model, facilities lacking Xpert testing capabilities rely on sample transfer to those with Xpert.
- **District-level Decentralization of Xpert:** This scenario extends Xpert testing to all 266 district hospitals (additional 96 GeneXpert instrument), bringing the total number of Xpert-equipped facilities to 266. This reduces the need for sample transport for patients accessing care at these larger, district-level facilities. The 30 districts without designated district-level facilities were not included as potential Xpert sites in this scenario.
- **Full Decentralization of Xpert:** This scenario represents Xpert testing availability at all 703 simulated facilities that reported at least one TB case in the years of interest. This eliminates the necessity for sample transportation and the associated risk of samples remaining untested. This model represents an idealized configuration to establish a ceiling on diagnostic gains that could be realized with increasing testing deployment across healthcare systems.

# Simulation Projections & Sensitivity Analysis

## Projected Outcomes Under Simulated Scenarios

Table E: Projected outcomes under simulated scenarios. Simulated outcomes were projected for the baseline scenario, which reflected the placement of Xpert instruments in Ghana as of 2023, as well as for two experimental scenarios: district-level decentralization and full decentralization of Xpert. The values represent the median [95% uncertainty range] outcomes per 1 million population, derived from 1,000 posterior simulations. The projected changes between the decentralized scenarios and the baseline are reported as the average annual change in outcomes, percentage change relative to baseline, and total change relative to baseline. Negative values indicate a reduction in the outcome under the decentralized scenarios compared to the baseline.

|  | Projected outcomes in 2030 per 1 million population | | | District-Level Decentralized Xpert vs Baseline Scenario (2023-2030) | | | Full Decentralized Xpert vs Baseline Scenario (2023-2030) | | |
| --- | --- | --- | --- | --- | --- | --- | --- | --- | --- |
| scenario | Baseline Xpert placement | District-level Xpert decentralization | Full Xpert decentralization | Annual change | % change | Total change | Annual change | % change | Total change |
| Number of patients presenting to care | 4300 [3900 – 4700] | 4300 [3800 – 4700] | 4300 [3800 – 4700] | -4 [-14 – -0.72] | -0.09% [-0.26 – -0.02%] | -36 [-110 – -6] | -19 [-57 –  -5] | -0.38% [-1 – -0.11%] | -150 [-460 – -43] |
| Number of samples tested in-house | 1300 [1200 – 1400] | 2000 [1800 – 2200] | 4300 [3800 – 4700] | 800 [720 – 880] | 53% [52 – 53%] | 6400 [5700 – 7000] | 3400 [3000 – 3700] | 220% [220 – 220%] | 27000 [24000 – 30000] |
| Number of samples sent out for testing | 3000 [2700 – 3300] | 2300 [2100 – 2500] | 0 [0 – 0] | -800 [-880 – -720] | -24% [-24 – -23%] | -6400 [-7100 – -5700] | -3400 [-3700 – -3100] | -100% [-100 – -100%] | -27000 [-30000 – -24000] |
| Proportion of samples sent out for testing | 0.7 [0.69 – 0.7] | 0.53 [0.53 – 0.54] | 0 [0 – 0] | 0 [0 – 0] | -23% [-24 – -23%] | -0.03 [-0.04 – -0.03] | -0.02 [-0.02 – -0.02] | -100% [-100 – -100%] | -0.15 [-0.15 – -0.15] |
| Number of samples remaining untested | 1100 [640 – 1600] | 870 [490 – 1300] | 0 [0 – 0] | -300 [-440 – -170] | -24% [-24 – -23%] | -2400 [-3500 – -1400] | -1300 [-1900 – -730] | -100% [-100 – -100%] | -10000 [-15000 – -5900] |
| Proportion of all collected samples remaining untested | 0.26 [0.16 – 0.36] | 0.2 [0.12 – 0.27] | 0 [0 – 0] | 0 [0 – 0] | -23% [-24 – -23%] | -0.01 [-0.02 – -0.01] | -0.01 [-0.01 – 0] | -100% [-100 – -100%] | -0.06 [-0.08 – -0.03] |
| Proportion of sent-out samples remaining untested | 0.38 [0.23 – 0.51] | 0.38 [0.23 – 0.51] | N/A | 0 [0 – 0] | 0.02% [-0.74 – 0.75%] | 0 [0 – 0] | NA [NA – NA] | NA% [NA – NA%] | NA [NA – NA] |
| Number of Xpert tests performed | 3200 [2900 – 3500] | 3400 [3200 – 3700] | 4300 [3800 – 4700] | 300 [170 – 430] | 8% [4 – 13%] | 2400 [1400 – 3500] | 1300 [730 – 1800] | 36% [19 – 54%] | 10000 [5800 – 15000] |
| Number of positive Xpert tests | 180 [140 – 210] | 190 [150 – 230] | 230 [180 – 280] | 13 [7 – 23] | 7% [3 – 11%] | 110 [56 – 180] | 56 [31 – 95] | 28% [15 – 47%] | 450 [250 – 760] |
| Proportion of positive Xpert tests | 0.06 [0.04 – 0.07] | 0.05 [0.04 – 0.07] | 0.05 [0.04 – 0.06] | 0 [0 – 0] | -1% [-4 – 0.53%] | 0 [0 – 0] | 0 [0 – 0] | -4% [-12 – -0.22%] | 0 [0 – 0] |
| Number of TB infections | 250 [220 – 270] | 250 [220 – 280] | 250 [200 – 310] | 2 [-12 – 15] | 0.63% [-4 – 5%] | 14 [-100 – 120] | 8 [-54 – 63] | 3% [-19 – 23%] | 62 [-430 – 500] |
| Number of TB diagnoses (excluding false positive diagnoses) | 160 [120 – 210] | 170 [130 – 220] | 190 [150 – 250] | 7 [3 – 15] | 4% [1 – 8%] | 57 [21 – 120] | 30 [12 – 61] | 17% [6 – 37%] | 240 [95 – 490] |
| Number of false TB diagnoses (patients without TB) | 85 [41 – 130] | 79 [42 – 120] | 63 [31 – 110] | -6 [-19 – 5] | -6% [-16 – 7%] | -44 [-150 – 41] | -22 [-80 – 19] | -24% [-64 – 27%] | -180 [-640 – 150] |
| Diagnosis accuracy (PPV) | 0.65 [0.51 – 0.83] | 0.68 [0.54 – 0.83] | 0.75 [0.62 – 0.87] | 0 [0 – 0] | 3% [-0.79 – 9%] | 0 [0 – 0.01] | 0 [0 – 0.01] | 14% [-2 – 47%] | 0.02 [0 – 0.05] |
| Proportion of TB diagnosis made clinically (without an associated positive Xpert result) | 0.33 [0.22 – 0.46] | 0.27 [0.19 – 0.39] | 0.1 [0.03 – 0.17] | 0 [0 – 0] | -17% [-22 – -11%] | -0.01 [-0.02 – -0.01] | -0.01 [-0.01 – 0] | -69% [-91 – -45%] | -0.05 [-0.08 – -0.02] |
| Number of clinical diagnoses (TB diagnoses without an associated positive Xpert result) | 80 [54 – 120] | 68 [45 – 97] | 24 [8 – 46] | -15 [-27 – -6] | -16% [-22 – -8%] | -120 [-220 – -50] | -63 [-110 – -28] | -68% [-91 – -37%] | -500 [-900 – -230] |
| Number of correct clinical diagnoses (patients with TB) | 28 [5 – 59] | 22 [5 – 47] | 4 [0.3 – 11] | -6 [-14 – -0.18] | -20% [-24 – -3%] | -50 [-110 – -1] | -27 [-62 – -0.74] | -84% [-99 – -12%] | -210 [-490 – -6] |
| Number of incorrect clinical diagnoses (patients without TB) | 52 [18 – 94] | 46 [17 – 77] | 20 [4 – 40] | -8 [-22 – 1] | -14% [-23 – 4%] | -66 [-170 – 9] | -34 [-91 – 4] | -56% [-92 – 14%] | -270 [-730 – 33] |
| Clinical diagnosis accuracy (PPV) | 0.35 [0.06 – 0.74] | 0.33 [0.06 – 0.7] | 0.18 [0.01 – 0.6] | 0 [0 – 0] | -4% [-12 – 16%] | 0 [-0.01 – 0] | 0 [-0.01 – 0.01] | -46% [-96 – 190%] | -0.03 [-0.12 – 0.07] |
| Proportion of notified TB patients starting TB treatment | 0.85 [0.79 – 0.89] | 0.85 [0.8 – 0.9] | 0.86 [0.8 – 0.9] | 0 [0 – 0] | 0.19% [-0.33 – 0.8%] | 0 [0 – 0] | 0 [0 – 0] | 0.71% [-0.02 – 2%] | 0 [0 – 0] |
| Number of diagnosed patients failing to initiate treatment | 54 [40 – 76] | 56 [42 – 79] | 62 [45 – 94] | 2 [-0.74 – 6] | 4% [-1 – 9%] | 17 [-6 – 50] | 9 [-2 – 25] | 15% [-3 – 37%] | 72 [-14 – 200] |
| Number of persons starting TB treatment | 210 [190 – 230] | 210 [190 – 240] | 210 [170 – 270] | 2 [-10 – 13] | 0.8% [-4 – 6%] | 15 [-81 – 110] | 8 [-43 – 55] | 4% [-18 – 24%] | 66 [-350 – 440] |
| Number of persons with TB starting TB treatment | 140 [110 – 180] | 150 [110 – 190] | 160 [130 – 220] | 6 [2 – 13] | 4% [2 – 9%] | 51 [20 – 100] | 27 [11 – 54] | 17% [6 – 37%] | 220 [91 – 430] |
| Number of persons without TB starting TB treatment | 69 [33 – 100] | 65 [34 – 95] | 51 [24 – 85] | -5 [-16 – 4] | -6% [-16 – 7%] | -36 [-120 – 33] | -18 [-66 – 16] | -23% [-64 – 27%] | -140 [-530 – 130] |
| Proportion of treatments initiated by patients without TB | 0.33 [0.16 – 0.48] | 0.31 [0.16 – 0.44] | 0.24 [0.13 – 0.37] | 0 [0 – 0] | -6% [-13 – 3%] | 0 [-0.01 – 0] | 0 [-0.01 – 0] | -27% [-57 – 8%] | -0.02 [-0.05 – 0] |
| Number of incomplete treatments | 10 [2 – 26] | 10 [2 – 26] | 10 [2 – 27] | 0.07 [-0.87 – 1] | 0.86% [-7 – 9%] | 0.57 [-7 – 9] | 0.24 [-3 – 4] | 2% [-18 – 24%] | 2 [-20 – 30] |
| Number of persons with TB cured in treatment | 120 [84 – 160] | 130 [88 – 170] | 140 [100 – 190] | 5 [2 – 11] | 4% [1 – 9%] | 43 [16 – 86] | 23 [10 – 46] | 17% [6 – 36%] | 180 [78 – 370] |
| Number of TB-related deaths | 69 [36 – 120] | 64 [34 – 110] | 47 [23 – 89] | -5 [-11 – -2] | -7% [-11 – -3%] | -43 [-85 – -16] | -23 [-46 – -10] | -29% [-48 – -14%] | -180 [-370 – -77] |
| Number of patients failing to receive a test, initiate treatment or complete treatment | 1200 [710 – 1700] | 930 [560 – 1300] | 73 [51 – 110] | -300 [-430 – -170] | -22% [-23 – -21%] | -2400 [-3500 – -1400] | -1300 [-1800 – -730] | -94% [-96 – -89%] | -10000 [-15000 – -5800] |
| Number of TB patients failing to receive a test, initiate treatment or complete treatment | 93 [58 – 150] | 80 [52 – 120] | 34 [22 – 58] | -15 [-27 – -8] | -15% [-18 – -10%] | -120 [-220 – -63] | -65 [-120 – -35] | -62% [-75 – -45%] | -520 [-930 – -280] |

## Sensitivity Analysis Results


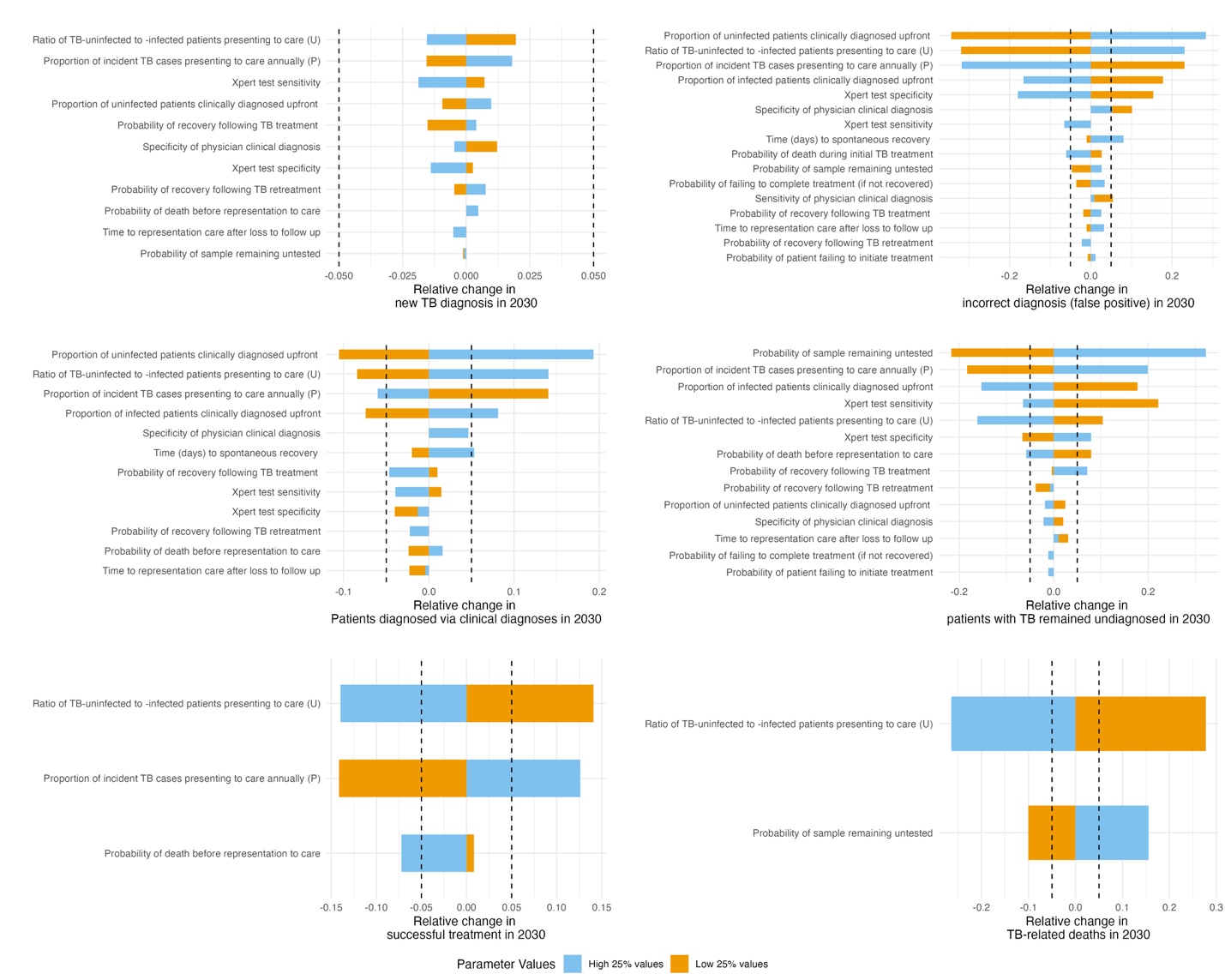
Figure F: One-way sensitivity analysis of parameter inputs to primary outcomes in the baseline scenario. For those parameters most strongly associated with the primary outcome (absolute value of partial rank correlation coefficient, PRCC >0.1), the subset of simulations with that parameter in the highest quartile (blue) is compared against simulations with that parameter in the lowest quartile (orange). The primary outcome of interest is the relative change in projected outcome at the baseline scenario in year 2030 (e.g., 1% represents a 1% increase in projected outcome compared to the primary analysis). Dashed lines mark a threshold of +/-5% to identify differences of potential epidemiologic importance.


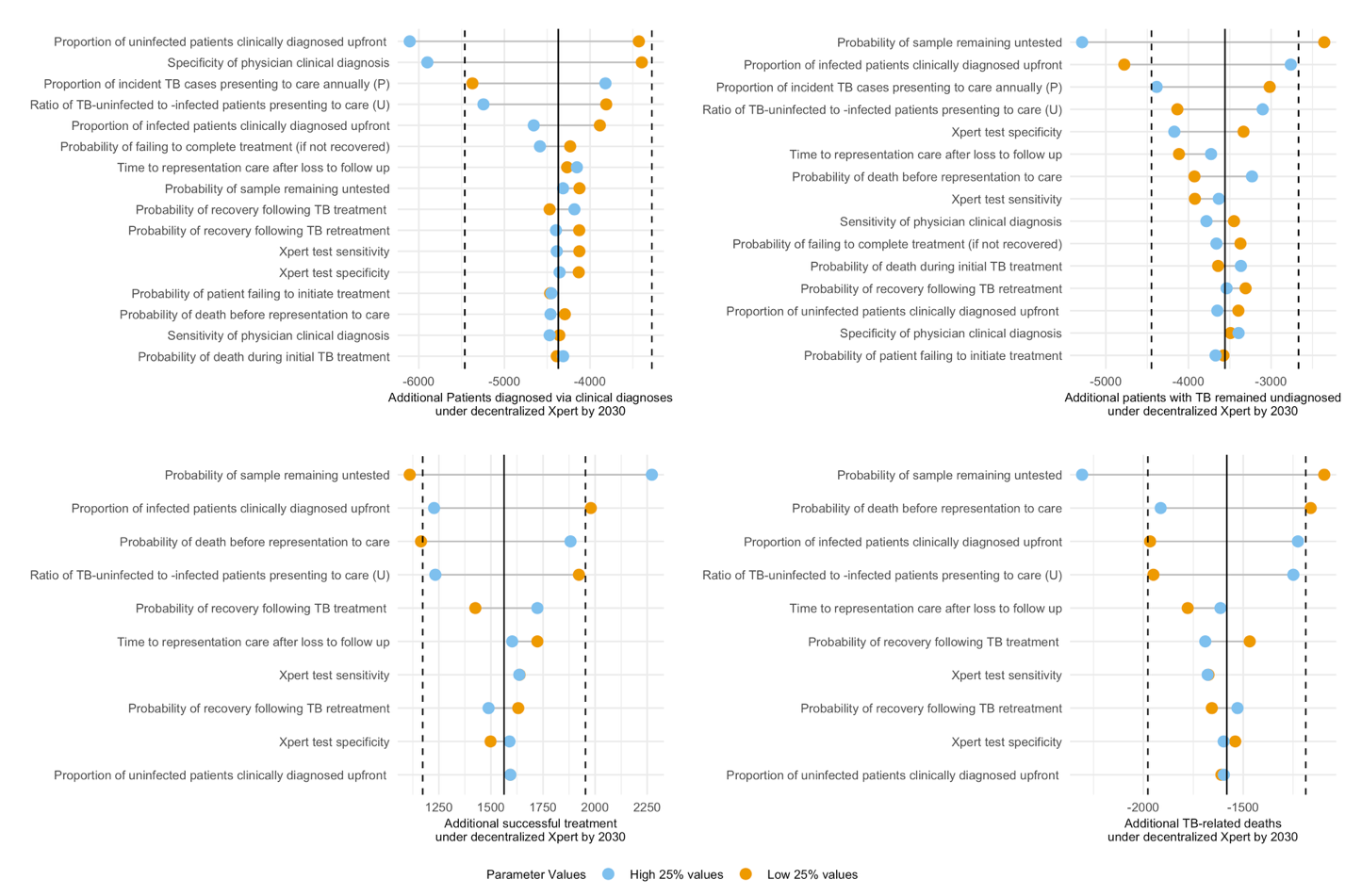


Figure G: One-way sensitivity analysis of parameter inputs on the impact of the district-level decentralized Xpert scenario on primary outcomes. For those parameters most strongly associated with the primary outcome (absolute value of partial rank correlation coefficient, PRCC >0.1), the subset of simulations with that parameter in the highest quartile (in blue) is compared against simulations with that parameter in the lowest quartile (in orange). The outcomes are calculated as the impact of district-level decentralized Xpert compared to the baseline scenario by year 2030. Solid vertical lines mark the projected impact in the primary analysis, and dashed lines mark a threshold of +/-25% to identify differences of potential epidemiological importance.

# Data Limitations

We observed discrepancies between TB cases reported in Ghana’s DHIMSII and those reported by the World Health Organization (WHO). This variation is largely driven by differences in data collection systems and reporting practices over time.

**A. Differences in Case Notification in WHO and DHIMSII Case Registration:** Initially, the National Tuberculosis Programme (NTP) reported cases to the WHO using manual Excel-based data collection tools. With the introduction of DHIMSII in 2017, facilities were required to begin entering TB case notifications directly into the system. However, the NTP continued using the Excel-based reports for WHO submissions until 2020 to ensure data quality and completeness in DHIMS-II. This transition accounts for the initial discrepancies observed between WHO-reported data and DHIMSII registration data in year 2018 during which case notification count in DHIMSII registration fell short by 13% compared to WHO's.

**B. Differences in Case Notification in DHIMSII Case Registration vs DHIMSII TB Screening Tool:** The TB screening tool was introduced in 2013, initially rolled out to 113 facilities (primarily hospitals and higher-level facilities) where task-shifting officers were employed to enhance screening activities. Although the program later scaled up the tool to all facilities, data entry practices continue to vary. Facilities that received early training tend to enter TB cases directly into the DHIMSII case registration module, often bypassing duplication on the screening tool, which leads to underrepresentation in screening tool records. Additionally, cases referred by civil society organizations, private clinics, and smaller facilities are typically recorded only in the DHIMSII case registration module, without being captured in the screening tool. This practices contribute to inconsistencies between data sources. In total, the reported case notifications in the TB screening tool falls approximately 30% lower than figures reported by the WHO between 2018 and 2022.

Due to lack of detailed information on the specific locations or facilities where this underreporting occurred, we chose not to incorporate this discrepancy directly into the model. Instead, we calibrated the model based the DHIMSII TB screening data, as that data source was unique in detailing the diagnostic and care cascade – including number screened for TB, number of presumed TB, number tested for TB, number diagnosed with TB, and number initiated on TB treatment. We assumed the ratios between steps in this cascade were accurate despite missingness of total numbers (that facilities not contributing to TB diagnosis data were also missing entries for the other steps in the cascade). This implies that facilities not reporting TB diagnoses were likely also missing data for earlier steps in the cascade, preserving internal consistency with the data.

# References

1. Nyonator F, Ofosu A, Osei D. District health information management system DHIMS-II: the data challenge for Ghana health service. Accra: Policy Planning Monitoring and Evaluation Division, Ghana Health Service. 2013.

2. Nkrumah B, editor Optimizing Ghana’s TB And HIV Diagnostic Networks

For Improved Access, Efficiency, And Reduced Cost:

Findings From A National Diagnostic Network

Optimization Analysis. African Society for Laboratory Medicine (ASLM); 2023.

3. AccessMod 5.6.0. World Health Organization, ; 2023.

4. Environmental Systems Research Institute (Esri). ArcGIS Pro (Version 3.2.0) 2024 [Available from: <https://www.esri.com/en-us/arcgis/products/arcgis-pro>.

5. WorldPop. Ghana 100m Population. . DOI: 10.5258/SOTON/WP00098 Alpha version 2010, 2014, and 2015 estimates of numbers of people per grid square, with national totals adjusted to match UN population division estimates (<http://esa.un.org/wpp/>) and 2010 and 2015 estimates of numbers of people per grid square remaining unadjusted2013 [Available from: [www.worldpop.org](file:///Users/Trinity/Dropbox/Ghana%20Lab%20Network%20R01/ModelDesign/ABM/InProgress/TB/Drafts/PlosGPH/resubmission/www.worldpop.org)

6. Macrotrends LLC. macrotrends.net 2024 [Available from: <www.https://macrotrends.net>.

7. Tuberculosis profile: Ghana [Internet]. World Health Organization. 2022 [cited 06/01/2024]. Available from: <https://worldhealthorg.shinyapps.io/tb_profiles/?_inputs_&entity_type=%22country%22&iso2=%22AF%22&lan=%22EN%22>.

8. Ghana Population 1950-2024 [Internet]. 2010-2024 Macrotrends LLC. 2023 [cited 06/01/2024]. Available from: <https://www.macrotrends.net/global-metrics/countries/GHA/ghana/population>.

9. World Health Organization (WHO). Tuberculosis profile: Ghana. 2022.

10. Theron G, Zijenah L, Chanda D, Clowes P, Rachow A, Lesosky M, et al. Feasibility, accuracy, and clinical effect of point-of-care Xpert MTB/RIF testing for tuberculosis in primary-care settings in Africa: a multicentre, randomised, controlled trial. The Lancet. 2014;383(9915):424-35.

11. MacPherson P, Houben RM, Glynn JR, Corbett EL, Kranzer K. Pre-treatment loss to follow-up in tuberculosis patients in low-and lower-middle-income countries and high-burden countries: a systematic review and meta-analysis. Bulletin of the World Health Organization. 2013;92:126-38.

12. Zifodya JS, Schiller I, Dendukuri N, Tollefson D, Schumacher SG, Ochodo EA, et al. Xpert MTB/RIF and Xpert MTB/RIF Ultra for pulmonary tuberculosis and rifampicin resistance in adults. Cochrane Database of Systematic Reviews. 2019(6).
